# Bioprocessing strategies for enhanced probiotic extracellular vesicle production: culture condition modulation

**DOI:** 10.3389/fbioe.2024.1441552

**Published:** 2024-08-30

**Authors:** Qingyu Lei, Shiva Kamini Divakarla, Tristrom Winsley, Shaun Roux, Wojciech Chrzanowski

**Affiliations:** ^1^ Sydney Pharmacy School, Faculty of Medicine and Health, The University of Sydney, Camperdown, VIC, Australia; ^2^ BiomeCentric PTY LTD., Molendinar, QLD, Australia; ^3^ Department of Laboratory Medicine, Division of Biomolecular and Cellular Medicine, Division of Clinical Immunology, Karolinska Institute, Stockholm, Sweden; ^4^ Division of Biomedical Engineering, Department of Materials Science and Engineering, Uppsala University, Uppsala, Sweden

**Keywords:** extracellular vesicles, postbiotics, probiotics, biomanufacturing, microenvironment, *Lactobacillus rhamnosus*

## Abstract

Probiotic extracellular vesicles are biochemically active structures responsible for biological effects elicited by probiotic bacteria. *Lactobacillus spp*., which are abundant in the human body (e.g., gut), are known to have anti-inflammatory and antimicrobial properties, and are commonly used in food products, supplements, and in discovery research. There is increasing evidence that *Lactobacillus*–derived extracellular vesicles (LREVs) have potent immunomodulatory capacity that is superior to probiotics themselves. However, key mechanistic insights into the process that controls production and thus, the function of LREVs, are lacking. Currently, it is unknown how the probiotic culture microenvironment orchestrates the type, yield and function of LREVs. Here, we investigated how multifactor modulation of the biomanufacturing process controls the yield and biological functionality of the LREVs. To achieve this, we selected *Lacticaseibacillus rhamnosus* as the candidate probiotic, initially cultivated under traditional culture conditions, i.e., 100% broth concentration and pH 5.5. Subsequently, we systematically modified the culture conditions of the probiotic by adjusting three critical process parameters: (1) culture medium pH (pH 3.5, 5.5 and 7.5), (2) growth time (48 and 72 h), and (3) broth concentration (50% and 10% of original broth concentration). EVs were then isolated separately from each condition. The critical quality attributes (CQA) of LREVs, including physical characteristics (size, distribution, concentration) and biological composition (protein, carbohydrate, lipid), were analysed. Functional impacts of LREVs on human epidermal keratinocytes and *Staphylococcus aureus* were also assessed as CQA. Our findings show that the production of LREVs is influenced by environmental stresses induced by the culture conditions. Factors like broth concentration, pH levels, and growth time significantly impact stress levels in *L. rhamnosus*, affecting both the production and composition of LREVs. Additionally, we have observed that LREVs are non-toxicity for keratinocytes, the major cell type of the epidermis, and possess antimicrobial properties against *S. aureus*, a common human skin pathogen. These properties are prerequisites for the potential application of EVs to treat skin conditions, including infected wounds. However, the functionality of LREVs depends on the culture conditions and stress levels experienced by *L. rhamnosus* during production. Understanding this relationship between the culture microenvironment, probiotic stress response, and LREV characteristics, can lead to the biomanufacturing of customised probiotic-derived EVs for various medical and industrial applications.

## Graphical Abstract

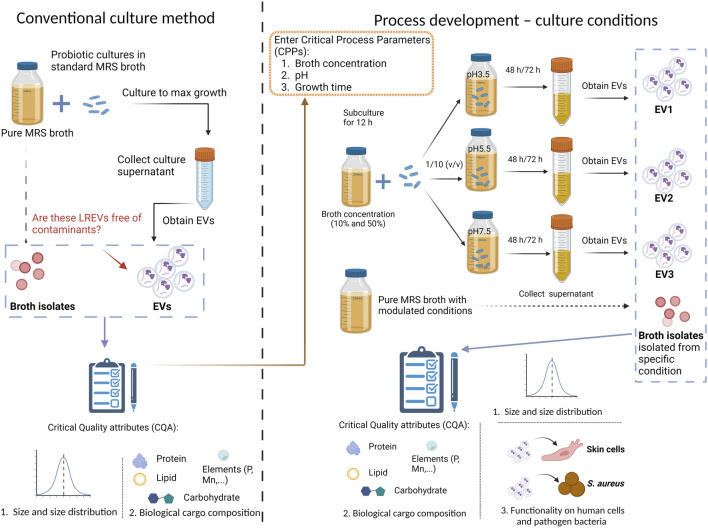

## Highlights

• Full-strength broth resulted in significant broth contaminants, affecting the production and purity of EVs.
• The modulation of culture conditions impact stress levels in *in Lacticaseibacillus rhamnosus,* affecting both the production and composition of LREVs.
• LREVs are safe for keratinocytes and effective against *S. aureus*. Safety and antimicrobial activity of EVs produced by L*. rhamnosus* depends on the culture conditions and stress levels.
• The modulation of the microenvironment, and probiotic stress response can improve the functionality of probiotic EVs that is the context of use-dependent.

## Introduction

Probiotics are a group of microbes, including bacteria and yeast, that can provide health benefits to the host when administered in sufficient quantities. They can stimulate or inhibit the activity of bacteria that are either beneficial or detrimental to the host’s health (Wang et al., 2022). Lactic acid bacteria (LAB) including genera such as *Lactobacillus*, *Bifidobacteria*, *Streptococcus*, *Pediococcus,* and *Leuconostoc*, represent the primary probiotic bacterial genera (Reuter, 1985). Evidence indicates that these bacteria may provide multiple health benefits, including improving the intestinal microbiota balance, and immunomodulatory capacity, reducing serum cholesterol, and even preventing cancer (Latif et al., 2023). It is crucial to note that these health benefits of probiotics are not only specific to the source, species, and strain but are also impacted by dietary habits, behavior, and the microenvironment in which probiotics are embedded (Cheng D. et al., 2019).

*Lactobacillus*, which are commonly isolated from the human gastrointestinal (GI) tract and from fermented foods, represents the genus of probiotic bacteria that is largest and most diverse, exhibiting beneficial effects on the host (Giraffa et al., 2010; Almonacid et al., 2017; Delanghe et al., 2021). For decades, *Lactobacillus* spp. have been used as a bacteriotherapy for the GI system and oral cavity, with effects on pathogen inhibition/exclusion and regulation of the immune response (Kopp-Hoolihan, 2001; Chugh et al., 2020). As an example, *Lactiplantibacillus plantarum* can inhibit the oral pathogen *Streptococcus mutans*, and reduce biofilm formation, suggesting the potential of using *L. plantarum* as the treatment of caries (Zhang et al., 2020). Another species like *Lacticaseibacillus rhamnosus* can increase the diversity of intestinal microbiota and modulate the balance of the GI system (Chen et al., 2019). Application of the strain *L. rhamnosus GG* has demonstrated that it can alleviate the effects of pro-inflammatory cytokines on epithelial barrier integrity and inflammation *in vitro* (Donato et al., 2010). However, our comprehension of probiotic function in the gut remains incomplete, leading to unpredictability in their functionality (Kechagia et al., 2013). Meanwhile, the efficacy of probiotics heavily relies on the timing of treatment and standardized dosage (Cheng D. et al., 2019). The challenges of unpredictability and standardization in probiotics have shifted current research focus towards postbiotics. Postbiotics, described as the “preparation of inanimate microorganisms and/or their components that confers a health benefit on the host” from ISAPP (Salminen et al., 2021), are implicated in driving the primary biological effects of probiotics.

While the specific pathways and key regulatory mechanisms behind the health benefits of probiotics remain largely unknown, it is evident that these health effects can be mediated, at least in part, by extracellular vesicles (EVs) derived from probiotics (Plaza-Diaz et al., 2019). EVs represent a heterogeneous population of cell-derived membranous vesicles, originating either from the endosomal compartment or the plasma membrane across all three domains of cellular life–Archaea, Bacteria, and Eukarya (van Niel et al., 2018; Gill et al., 2019). Probiotic EVs are recognized as a type of vectorial secretion (a component of postbiotics) and they are crucial mediators of intracellular signalling *via* the transfer of macromolecular cargoes, including nucleic acids, virulence factors, and cytoplasmic proteins (Chronopoulos and Kalluri, 2020). The roles of EVs, and in particular bacterial EVs, in promoting health and in causing various pathologies, whether through bacterial–bacterial or bacterial–host interactions, are becoming increasingly evident. For instance, EVs produced from *L. plantarum* exhibit protective effects on hosts and show promise in treating pathogens with antimicrobial-resistant properties (Li et al., 2017). Additionally, EVs derived from *Lacticaseibacillus paracasei* can reduce lipopolysaccharide-induced inflammation, playing a significant role in maintaining colorectal homeostasis in inflammation-mediated pathogenesis (Choi et al., 2020).

Despite the promising role of probiotics and their EVs in health applications, there is still a need for understanding of the mechanisms surrounding their influence upon the host and the effects of the surrounding microenvironment on EVs production (Macia et al., 2019). While previous studies have focused on optimizing isolation strategies (Watson et al., 2021; Castillo-Romero et al., 2023), they often overlook the impact of pre-isolation factors, such as the full-strength broth, on bacterial EV production and purity. These limitations not only hinder production efficiency and introduce contaminants into EV samples but also impact the effectiveness of EVs in their intended biological applications.

At present, there are limited studies related to the mechanism of biogenesis and release of probiotic EVs. However, strategies to increase EV secretion for both human cell-derived and bacterial EVs have been extensively investigated (Debbi et al., 2022; Erwin et al., 2023; Muñoz-Echeverri et al., 2024). These strategies include genetic modifications of the producer cells, environmental stressors, and biomechanical stimulation. Among these, the most commonly used culture-based strategies for enhancing bacterial EV production are: the modulation of pH, temperature, oxygen level, and nutrient depletion (Muñoz-Echeverri et al., 2024). For instance, an acidic pH of 5.3 increased EV release from *Francisella tularensis* by 3-fold compared to neutral conditions, while increasing the temperature to 42°C enhanced EV production by 4.75-fold compared to 37°C (Klimentova et al., 2019). Gerritzen et al. (2018) also showed that an increase in air saturation from 30% to 100% increased the OMV production by *Neisseria meningitidis* by 3-fold. Additionally, depleting cysteine in the culture of *N. meningitidis* increased OMV production, which was shown to be culture time-dependent (van de Waterbeemd et al., 2013). Collectively, modulating culture conditions is an effective strategy to boost the EV production and modify their properties.

Therefore, in our studies we applied culture-based strategies and investigated their role in the production of EVs from probiotics. Given the numerous factors that can affect EV production and purity, it is not feasible to evaluate all parameters in a single study. Instead, we focused on modulating conditions relevant to human environments, namely, pH and nutrient availability, while keeping temperature, oxygen levels, and osmotic stress constant to avoid the confounding effects of other factors. Identifying these factors is essential for determining the critical process parameters (CPPs) that influence the critical quality attributes (CQAs) of EVs, ensuring the development of reproducible and clinically viable EV-based products (Costa et al., 2023). Previous research has shown that CPPs, including environmental pH stress (Nakase et al., 2021), serum starvation/culture media composition (Guerreiro et al., 2018), and modulation of cell growth time, significantly impact the yield and purity of bacterial EVs. These established CPPs can be extended to probiotics and probiotic EVs given that factors like pH (Ratzke and Gore, 2018), growth time (Terpou et al., 2019), and serum presence (Gutiérrez et al., 2016) are essential in the growth, performance and functionality of probiotics. Consequently, these parameters should play key roles in the secretion and properties of probiotic EVs.

Here, we utilized *L. rhamnosus* as the probiotic candidate, cultivating it under conventional conditions. We then systematically adjusted three critical process parameters (CPPs): environmental pH, probiotic growth time, and broth concentration to optimize culture conditions. EVs were isolated from each condition, and their critical quality attributes (CQAs) were evaluated, including physical characteristics (size, distribution, concentration), and biological composition (protein, polysaccharide, and lipid content). Furthermore, we examined the effects of LREVs on human epidermal keratinocytes and *Staphylococcus aureus* as CQAs, evaluating their impact on both human cells and pathogenic bacteria.

## Method

### Probiotic culture using conventional culture method

*L. rhamnosus* was cultured in de Man, Rogosa, and Sharpe medium (MRS broth, Oxoid, Thermo Fisher SCIENTIFIC, United States of America) with standard broth concentration (100%) and a pH adjusted to 5.5. The culture was then incubated at 37°C (±1°C) for 12 h under static culture conditions in the incubator sharker (SPH-103B, SHIPING Temperature, China). Subsequently, the culture was scaled up to 1 L using a 10% inoculation from the starting culture and maintained at 37°C (±1°C) for 72 h. Bacterial growth was monitored by measuring the optical density at 600 nm (OD_600_) using plate reader. Cell morphology was imaged using 3D Cell Explorer (Nanolive SA, Switzerland) and Atomic Force Microscopy (AFM, Bruker, United States of America). The culture was maintained for 72 h for EV isolation.

### Probiotic culture using modulated culture conditions

To modulate the culture conditions of bacteria, three key variables were chosen: pH level, growth time, and broth concentration, with the aim of investigating their impact on both bacterial growth and EV production. The experiment involved three pH levels (i) pH 3.5, pH 5.5, and pH 7.5, two growth times (ii) 48 and 72 h, and two broth concentrations (iii) 50% of the standard broth concentration and 10% of the standard broth concentration.

*L. rhamnosus* was cultured in de Man, Rogosa, and Sharpe medium (MRS broth, Oxoid, Thermo Fisher SCIENTIFIC, United States of America) with reduced broth concentration 10%, 50% with an initial pH of 5.5. The culture was then incubated at 37°C (±1°C) for 12 h under static culture conditions. Subsequently, the culture was scaled up by transferring 100 mL of subculture into 900 mL of fresh MRS broth with designed pH and broth concentration and maintained for specific growth time. Bacterial growth was monitored by measurement of OD_600_. Cell morphology was imaged using 3D Cell Explorer as described above.

### Probiotic EV isolation

To obtain 100 mL of probiotic ferments, conventional culture conditions (Cond.org in Table 1) and modulated culture conditions (Cond.1-12 in Table 1) were utilized, followed by double centrifugation at 8,000 *g* for 10 min to eliminate bacteria. Subsequently, the supernatant from the final centrifugation was filtered through a 0.45 µm SFCA membrane (Corning^®^, United States of America) to remove cell debris. LREV isolation, concentration, and buffer exchange with 0.01 M HEPES buffer were carried out using a TFF-EASY™ system (HansaBioMed Life Sciences, Tallinn, Estonia).

**TABLE 1:** The culture and isolation conditions (Cond.) for LREV and broth isolates, including both conventional (Cond.org) and modulated methods (Condi.1-12).

Conventional culture			Modulated culture						
Broth		100%	Broth	50%			10%		
pH		pH 5.5	pH	pH 3.5	pH 5.5	pH 7.5	pH 3.5	pH 5.5	pH 7.5
Growth time	72 h	Cond.org	48 h	Cond.1	Cond.2	Cond.3	Cond.7	Cond.8	Cond.9
			72 h	Cond.4	Cond.5	Cond.6	Cond.10	Cond.11	Cond.12

Control samples, consisting of 100 mL of pure MRS broth without probiotics, were also prepared, following either conventional culture conditions (Cond.org) or modulated conditions with specific pH and concentration adjustments (Condi.4-6 and Condi.10-12 as listed in Table 1). These control samples underwent the same isolation steps and procedures as the probiotic samples and were considered as broth isolates for comparative analysis.

### Physicochemical characterisation of probiotic EVs using NanoFCM

#### Characterisation of size, size distribution and concentration

To characterise LREVs, we determined three LREVs quality attributes (QA): size, size distribution, and concentration. QAs were measured using a Nano-Flow analyser (NanoFCM, Xiamen, China). The calibration was done using NanoFCM Quality Control Nanospheres (NanoFCM, Xiamen, China) for laser alignment and concentration, and NanoFCM Silica Nanospheres Cocktail #1(NanoFCM, Xiamen, China) for size. Prior to sample measurement, a PBS blank was used as a control to remove the background. Next, samples were then loaded and size, size distribution, and concentration were determined through Scatter mode.

#### Stability assessment

For the stability study we selected one type of EVs: LREVs, 50% broth concentration, pH 5.5, and 72-h culture time. The stability of EVs was assessed by measuring size, size distribution and zeta potential for samples stored at 4°C in PBS for 4 weeks using single nanoparticle measurement system Exoid–Tuneable Resistive Pulse Sensing (Exoid, Izon Science, New Zealand). The advantage of using Exoid is that it provides a broader measurement range (50–330 nm using NP100 Nanopores) and includes zeta potential analysis using nanopore technology, offering detailed particle-by-particle analysis to determine aggregation/degradation of nanoparticles, here LREVs (Supplementary Materials).

#### Purity assessment

Two additional studies were used to confirm LREVs purity: (1) Triton X-100 study and (2) exosome labelling using PKH 67.

##### Triton X-100 study

Non-ionic surfactant Triton X-100 was used to lyse the phospholipid bilayer of LREVs for LREVs identification. After measuring the size and size distribution by NanoFCM, EV sample was permeabilized with 0.1% (v/v) Triton X-100 (Sigma-Aldrich, US). NanoFCM was then used to measure change of the particle counts before and after Triton X-100 treatment to confirm the purity of EV samples.

##### Exosome labelling using PKH 67

To improve LREVs visualization in NanoFCM for quantitative assessment, PKH lipophilic membrane dyes were employed to stain both LREVs and broth isolates. The PKH67 green-fluorescent cell linker kit (MINI67, Sigma-Aldrich, United States of America) was utilized for the staining procedure, following the manufacturer’s instructions. LREVs and broth isolates were diluted fivefold using Diluent C from the kit. Subsequently, PKH67 dye was applied to stain the lipid membrane for 4–5 min, followed by quenching with 1% BSA. To remove the unbound dye, the stained sample was passed through a 300 kDa NanoSep column (Nanosep™ centrifugal devices with Omega™ membrane 300K, 0.3 cm^2^, Cytiva, Sweden) and centrifuged at 4,000 *g* for 5 min. To minimize background fluorescence from the dye, the sample collected from the upper side of the column was washed three times using HEPES buffer at 4,000x g for 5 min each and then resuspended in HEPES buffer. All samples were further diluted fivefold to achieve a final dilution factor of 25, and the number of the fluorescently-labelled vesicles was determined using NanoFCM.

### Characterization of probiotic EVs’ biological compositions

#### BCA assay for protein content

To analyse the protein content of the sample, Pierce™ BCA Protein Assay Kit - Reducing Agent Compatible (23250, Thermo Scientific™, United States of America) was used according to the manufacturer’s protocol. Briefly, protein standards were prepared with various concentrations of BSA, to generate the standard curve correlating total protein concentrations with OD. Then, LREVs, and broth isolates control were added to a 96-well plate and mixed with compatibility reagent solution. The plate was incubated at 37°C for 15 min. Then, the working reagent was added to each well, after which the plate was incubated again at 37°C for 30 min. The plate was cooled to room temperature for 5 min and the absorbance of the standards and LREVs/broth isolates samples was measured at 560 nm using a plate reader (VICTOR^®^ Nivo™ Plate Readers, PerkinElmer, United States of America).

#### Phenol-sulfuric acid assay for carbohydrate content

We used the phenol-sulfuric acid assay to determine the amount of carbohydrates present in the LREVs sample and broth isolates control as previously described (Nielsen, 2010). Carbohydrate standards were prepared with various concentrations of glucose, to generate the standard curve correlating total carbohydrate concentrations with OD. 50 μL of the standard solution and 50 µL of the EV/broth samples were added separately into the 96 well plate. Then, 150 µL of 98% sulfuric acid (wt%) and 30 µL of 5% phenol (wt%) were added to each standard solution and sample and mixed in the 96-well plate. The plate was heated up at 100°C for 5 min and cooled down for 15 min to room temperature prior to reading. The absorbance was measured at 490 nm.

#### Sulfo-phospho-vanillin assay for lipid content

To analyse the total lipid content within LREVs and broth isolates, we used the sulfo-phospho-vanillin assay (SPVA) (McMahon et al., 2013). Lipid standards were prepared with various concentrations of cholesterol, dissolved in chloroform: methanol at a ratio of 2:1 to generate the standard curve correlating total lipid concentration with OD. Both standard and LREVs/broth isolates solutions were evaporated at 90°C for 30 min. Subsequently, 100 µL of 98% sulfuric acid (wt%) was added to the evaporated samples and standards, followed by heating at 90°C for 10 min. The heated samples were then cooled to room temperature for 15 min. Background absorbances were recorded by a plate reader at 550 nm. For colour development, 100 µL of phosphoric vanillin acid agent containing 0.2 mg of vanillin dissolved per 1 mL 17% phosphoric acid (wt%) was added. The final absorbance after colour development was measured at 550 nm.

#### Elemental analysis of probiotic bacteria and probiotic EVs using ICP-MS

To prepare the digested bulk LREVs sample, LREVs were lysed using 1% HNO_3_ (wt%) for elemental analysis. To compare the elemental composition between LREVs and source of origin, 1 mL of *L. rhamnosus* culture was obtained under specific conditions and at a particular time point. The culture was then centrifuged at 8,000 *g* for 10 min to pellet bacteria. The supernatant was removed, and the bacterial pellet was resuspended in 0.01 M HEPES buffer. Subsequently, it underwent further centrifugation at 8,000 *g* for 10 min to eliminate all broth interference. The washed bacterial pellets were digested using 70% HNO_3_ (wt%) and then further diluted to 1 mL for elemental analysis. By using argon plasma to atomize samples, ICP-MS (PerkinElmer, Nexion 2000; United States of America), a hyperbolic quadruple mass spectrometer, can determine the isotopic elemental concentration of LREVs and the real concentration can subsequently be ascertained using the appropriate elemental standards. To eliminate spectral interference, we selected and measured the concentration of the following elements ^43^Ca, ^31^P, ^59^Co, ^66^Zn, ^39^K, ^24^Mg, ^27^Al, and ^55^Mn, and the measurements were done on bulk digested samples.

### Biological assessment of probiotic EVs

#### Cell culture and cell toxicity study

The immortalized human keratinocyte cell line, HaCaT, was cultured in in DMEM culture medium, containing Dulbecco’s Modified Eagle’s Medium (DMEM medium-high glucose/e, Sigma-Aldrich), 10% Foetal Bovine Serum (FBS, Bovogen, Australia) and 1% PenStrep. Phosphate buffered saline (PBS, 1×, Sigma-Aldrich, US) was used for washing cells. TrypLE™ Express (Gibco™, US) was used as the dissociation reagent for adherent cells. All cell lines were maintained at 37°C and 5% CO_2_.

HaCaT cells were seeded in a 96-well cell culture plate (Corning^®^, United States of America) at a density of 5,000 cells per well. After incubation at 37°C and 5% CO_2_ for 24 h, the culture medium was replaced with two concentrations (10^4^ and 10^2^ EVs per cell) of LREVs (Condi.1-12 as listed in Table 1) diluted with DMEM culture medium. Broth isolates (Condi.4-6 and Condi.10-12 as listed in Table 1) were employed as the control treatment. The volume and concentration of the broth isolates were adjusted to correspond to the same dilution ratio used for the LREVs samples isolated from specific condition. Maintaining consistency in the treatment volume/dilution ratio allowed for the isolation and evaluation of the specific effects of LREVs on cell viability, comparing them to the baseline response observed in the control treatment. All treatments were maintained with cells for a duration of 72 h. Cell morphology and behaviour was recorded using an Incucyte^®^ S3 (Sartorius AG, Germany). Following the 72-h treatment, a CCK-8 solution (DOJINDO, Japan) was prepared using manufacturer’s protocol. Subsequently, all treatments and the medium in the wells were replaced by 100 μL of the CCK-8 working solution, and the cells were incubated with the working solution for 1 h at 37°C. Absorbance at 450 nm was measured with a plate reader after incubation. The percentage of cell viability for each treatment was presented relative to the control.

#### Minimum inhibitory concentration test (MIC)

*S. aureus* strains were used to determine the antimicrobial properties of LREVs and broth isolates. The strains were initially plated onto tryptone soy agar (TSA) plates and incubated overnight under aerobic conditions at 37°C. Single colonies from the agar plates were picked to inoculate 20 mL cultures in tryptone soy broth (TSB) medium (Thermo Scientific™, United States of America), adjusted to pH 7. The cultures were then allowed to grow for 16 h at 37°C with continuous shaking at 150 rpm. After the initial growth period, the bacterial suspension was diluted to 10%, and a sub-culture was performed for 2 h at 37°C with agitation at 150 rpm. Following this sub-culture, the bacterial culture was further diluted 1:10 in preparation for the growth assay.

All LREVs samples and broth isolate controls were included with the bacterial culture at various concentrations ranging from 0.625% to 5% (v/v). OD_600_ was measured to determine growth. A positive control, comprising non-treated bacterial suspension, was used as the reference for comparison. Results were reported in terms of percentage bacterial growth reduction, relative to the positive control. The experiment was performed in triplicate and the average values were used for data analysis.

#### Statistical analysis

Each group of experiments was repeated in triplicate, and three parallel samples were tested. The results were expressed as mean ± SD. At the same time, GraphPad software was used to carry out statistical analysis and significant analysis of the experimental data (*p* < 0.05).

## Results

### Growth and morphological characteristics of *L. rhamnosus*

**FIGURE 1:**
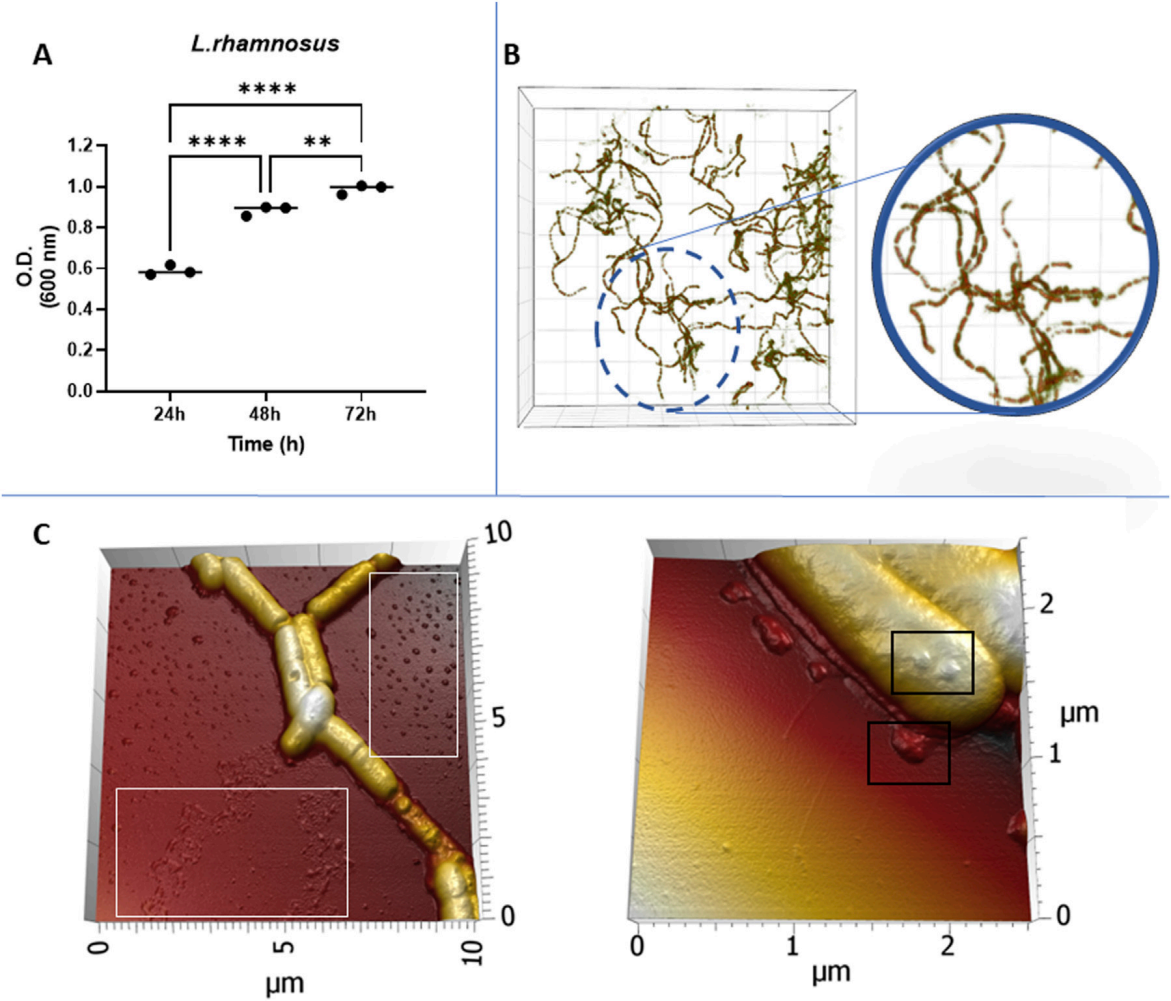
**(A)** Bacterial growth monitoring using optical density at 600 nm (OD_600_). **(B)** Visualization of bacterial morphology captured by Nanolive imaging. **(C)** Structural characteristics revealed by Atomic Force Microscopy (AFM) with vesicular-shaped particles in the background (White rectangles) and a budding behaviour on the membrane surface (Black rectangles).

To ensure an optimal culture condition for the production of EVs, we monitored the growth and morphology of *L. rhamnosus*. The analysis of the growth showed a fast-growing log phase occurring within the initial 48 h of cultivation. After this period, bacteria remained in stationary phase with a consistent and high microbe density at 72 h, indicating that the bacteria growth entered a stationary phase (Figure 1A). The morphology of *L. rhamnosus* displayed characteristic rod-shaped, long-chain structures, with a tendency to associate in intertwined clusters (Figure 1B). Detailed examination of the morphology of *L. rhamnosus* revealed vesicular-shaped particles in the background (Figure 1C-white rectangles) surrounding *L. rhamnosus*, and a notable tendency for budding vesicles on the membrane surface (Figure 1C-black rectangles) of *L. rhamnosus*.

### Physical and biological characteristics of EVs derived from *L. rhamnosus* (LREVs) cultured in conventional conditions

**FIGURE 2:**
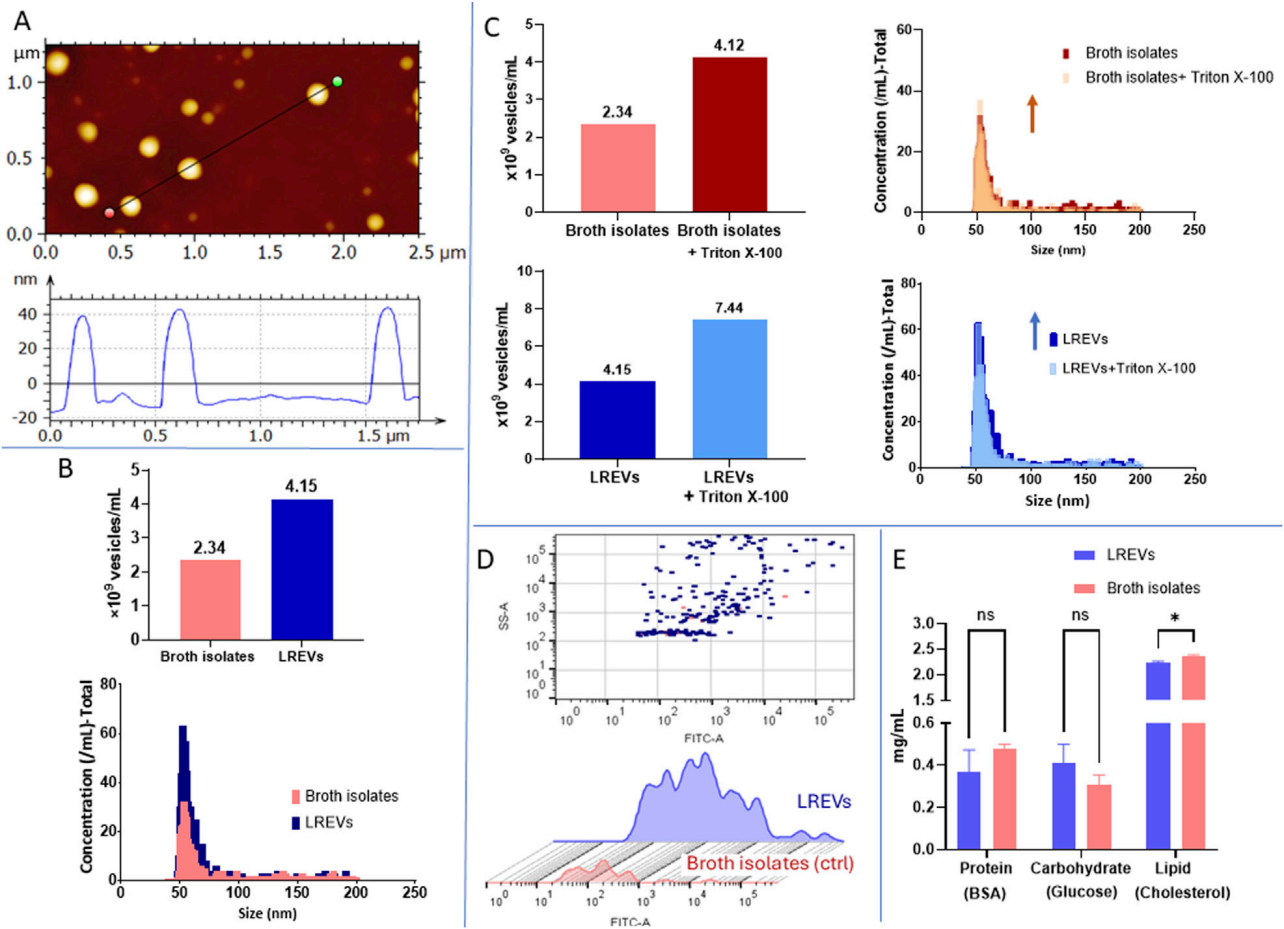
Physical and biological characteristics of EV derived from *L. rhamnosus* (LREVs) cultured in conventional condition. **(A)** the images include size and morphology of LREVs obtained from AFM. **(B)** the concentration, size, and size distribution obtained from NanoFCM for both LREVs and broth control. **(C)** the concentrations and size distribution of broth isolates LREVs before and after Triton X-100 treatment is presented. **(D)** the comparison of lipid-stained broth isolates and LREVs recorded by NanoFCM. **(E)** The comparation of protein, lipid and sugar composition of LREVs and broth isolates.

To assess the impact of conventional culture conditions, *i.e.*, standard broth concentration and pH 5.5, on the size and composition of LREVs, we conducted a comparative analysis of the physical characteristics of LREVs with a control group of broth isolates. The size and morphology assessment of LREVs revealed an average size of 60 nm with a spherical morphology (Figure 2A).

Using DLS, we determined that both LREVs and broth isolates showed a non-Gaussian size distribution with three dominating peaks: ∼20 nm (Size 1) to ∼200 nm (Size 2), and >4,000 nm (Size 3) (Supplementary Figure S1), These peaks displayed different intensity distribution weighted according to the scattering intensity of its respective particle fraction but displayed exhibited similar intensities across both LREVs and broth isolates. Statistical analysis showed no significant differences between LREVs and broth isolates for small size range (Size 1) and large size range (Size 3), with the only significant statistically significant for middle size range (Size 2).

Using NanoFCM, we determined that the concentrations of broth isolates and LREVs were 2.34 × 10^9^ EVs/mL and 4.15 × 10^9^ EVs/mL, respectively (Figure 2B). The size of LREVs and broth isolates was distributed between 40–200 nm. LREVs had a mean size of 58 ± 10 nm, while the mean size of broth isolates was 57 ± 9 nm, indicating that the size distribution of broth isolates and LREVs were not significant.

To assess the purity of LREVs (the number of LREVs in the EV preparations), we measured the concentration, size, and size distribution of both broth isolates and LREVs before and after Triton X-100 treatment (Figure 2C). A reduction in particle counts within LREVs indicates disruption of LREVs’ membrane integrity by Triton X-100, indicating the amount of LREVs, hence sample purity. Following Triton X-100 treatment, the concentration of particles in broth isolates increased by 75.9% compared to pre-treatment levels, with a mean size of 55.1 ± 7.6 nm. Similarly, there was a 77.6% increase in LREVs concentration post-treatment, with a mean size of 55.7 ± 9.2 nm. The increased concentration and size similarity between the broth isolates and LREVs before/after Triton X-100 treatment suggest that the isolated particles (initially assumed as LREVs) were not enveloped by a lipid bilayer that can be disrupted by Triton X-100, and these particles were rather contaminants derived from the broth.

To further study the presence of EVs in isolated samples, we labelled the EV membrane using the green-fluorescent dye PKH67. Using NanoFCM, we detected 29 stained vesicles within broth isolates and 227 stained vesicles within LREVs (Figure 2D). Although the LREVs exhibited a higher number of stained vesicles compared to the broth isolates, the proportion of these stained vesicles compared to the total number of LREVs present was less than 10%. This indicates that over 90% of the isolated vesicles were not considered EVs.

The protein, carbohydrate and lipid content of LREVs and broth isolates was evaluated (Figure 2E). The protein and carbohydrate content within LREVs and broth isolates were similar, with values of 0.37 and 0.48 mg/mL for protein, and 0.4 and 0.31 mg/mL for carbohydrate, respectively. No significant differences were observed in the protein and carbohydrate content between LREVs and broth isolates. Lipid analysis revealed high concentrations of lipid in both LREVs, and broth isolates compared to protein and carbohydrates. The lipid content in LREVs and broth isolates was measured at 2.2 mg/mL and 2.3 mg/mL, respectively, with only a difference of 5.6% between them. These results indicate that the protein, carbohydrate, and lipid compositions were comparable between LREVs and broth isolates, with broth isolates exhibiting a slightly higher lipid concentration.

**FIGURE 3:**
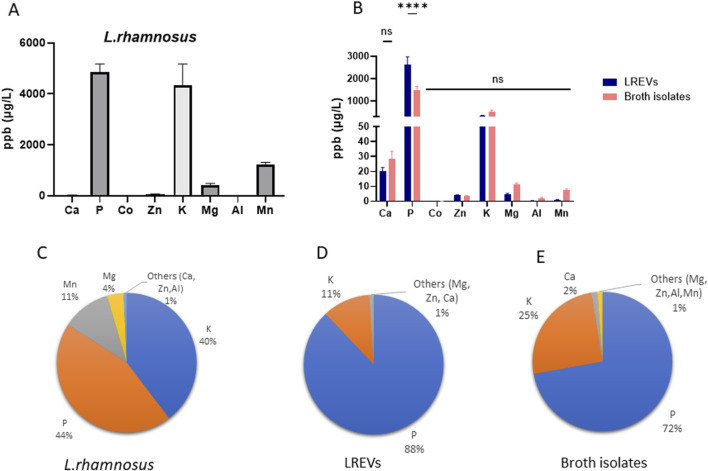
Elemental composition analysis of LREVs with their source of origin and microenvironment including the **(A)** elemental composition of *L. rhamnosus*, **(B)** the elemental composition and comparison of LREVs and broth isolates as well as **(C–E)** the elemental distribution of *L. rhamnosus*, LREVs and broth isolates. (ns: *p* > 0.05; ****: *p* ≤ 0.0001; mean ± SD).

To establish a connection between the elemental composition of LREVs and their source of origin, as well as and to compare isolated LREVs with their respective culture conditions, we conducted elemental analysis on *L. rhamnosus*, as well as on EVs obtained from *L. rhamnosus* (LREVs) and broth isolates (Figure 3).

The elemental analysis of *L. rhamnosus* and LREVs revealed differences in the elemental composition. *L. rhamnosus* contained P, K, Mg, and Mn, with P, K, and Mn showing the highest concentrations at 4,863 ppb, 4,333 ppb and 1,230 ppb respectively (Figure 3A). However, LREVs contained predominantly Ca, P, Zn, K, and Mg, with the highest relative concentrations observed for P and K at 2,633 ppb and 331 ppb, respectively (Figure 3B). These differences indicate that LREVs selectively carry specific types of elements.

When comparing the elemental composition between LREVs and broth isolates, there were no significant differences in Ca, Zn, K, Mg, Al, and Mn concentrations, but the concentration of P within broth isolates was 43% lower than that within LREVs (Figure 3B). A major elemental similarity between broth isolates and LREVs indicates the LREVs might acquire elemental characteristics from their surroundings except for lower P concentrations in broth isolates.

The analysis of the relative concentrations of elements and their contribution to the total amount of metal elements within *L. rhamnosus*, showed that LREVs and broth isolates the proportion of each element was different within *L. rhamnosus*, LREVs and broth isolates. In *L. rhamnosus*, we found that P represented 44% of the total amount of elements while K constituted 40%. Mn and Mg represented 12% and 4% of the total amount of elements respectively (Figure 3C). The remaining elements such as Ca, Zn and Al, collectively comprised only 1%. In LREVs, P represented 88% of the total amount of elements while K accounted for 11% (Figure 3D). The remaining elements, including Ca, Zn, and Mg, collectively constituted 1%. Similarly, in broth isolates, P represented 72% of the total amount of elements while K accounted for 25% (Figure 3E). Ca constituted 2% of the remaining elements, including Mg, Zn, Al and Mn, collectively constituting 1%. Collectively, P and K demonstrate as major contributors across all samples (*L. rhamnosus*, LREVs, and broth isolates), suggesting a connection between the source of origin and the surrounding microenvironment.

### The effect of modified culture conditions on physical and biological characteristics of EVs derived from *L. rhamnosus* (LREVs)

To modulate the pH level, we selected a middle value of pH 5.5 from the optimal pH for *lactobacilli* growth as the ideal condition and included two additional pH levels, 3.5 (relatively acidic) and 7.5 (relatively alkaline), for comparison. To address contamination issues and potential inhibition of EV production caused by full-strength broth, we used 50% and 10% broth concentrations. Additionally, to assess the impact of culture time on EV production, we selected two time points: 48 h and 72 h.

#### The results of the effect of modified culture conditions on growth of *L. rhamnosus*

We determined the effect of broth concentration and pH on the growth of *L. rhamnosus*. By reducing the broth concentrations to 50% (50% MRS), we observed that the growth of *L. rhamnosus* reached an optical density at 600 nm (OD) of 0.76 at 48 h and 0.83 at 72 h (pH 5.5) (Supplementary Figure S2A). Changing the pH to 3.5 resulted in a reduction in probiotic growth rate, with OD reaching 0.43 at 48 h and 0.40 at 72 h. When the pH was increased to 7.5, cells reached an OD of 0.82 at 48 h and an OD of 0.84 at 72 h. For *L. rhamnosus* cultured within 10% of broth concentration (10% MRS), we observed low cell densities under all conditions. When *L. rhamnosus* was cultured at pH 3.5, an OD reached only 0.02 at both time points: 48 and 72 h (Supplementary Figure S2B). Increasing the pH to 5.5 and 7.5 resulted in the improvement of *L. rhamnosus* growth, and ODs reached 0.18 and 0.24 respectively at both time points. These results show that bacterial density, hence their growth, is influenced by broth concentration and ph. The duration of culture has a lesser effect on bacterial density after 48 h, indicating that bacterial growth reaches the stationary phase.

The morphology of *L. rhamnosus* was notably affected by variations in broth concentrations and pH. When cultured in 50% MRS, *L. rhamnosus* exhibited typical rod-shaped, long-chain structures. However, when modifying the pH to 3.5, a small population of bacterial chains were observed, with some exhibiting a fuzzy edge (Supplementary Figure S2C, red arrows), suggesting that those were under stress. In contrast, at pH 5.5 and 7.5, *L. rhamnosus* formed longer bacterial chains with a larger population that tended to be entangled, particularly noticeable at pH 5.5 (Supplementary Figures S2D, E). When cultured in 10% MRS with a pH of 3.5, *L. rhamnosus* appeared to grow as rod chains (Supplementary Figure S2F, green arrows) with some forming clusters. In addition, we observed the presence of short/small fragments of the bacteria rods (Supplementary Figure S2F, blue arrows), which suggested that the structure and integrity of some bacteria were disrupted under these culture conditions. Under pH 5.5 and 7.5 conditions, *L. rhamnosus* tended to form relatively long rod chains, but some exhibited fuzzy edges (Supplementary Figures S2G, H, red arrows). These fuzzy appearances (red arrows) suggested a potential secretion of the extracellular matrix, implying that these bacteria were likely experiencing stressed conditions.

#### The results of the effect of modified culture conditions on size, size distribution and concentration of LREVs

To determine the effect of broth concentration (50% and 10%), pH (pH 3.5, pH 5.5 and pH 7.5) and growth time (48 and 72 h) of *L. rhamnosus* on LREVs, we measured the concentration, size, and size distribution of LREVs cultured under these different conditions.

**FIGURE 4:**
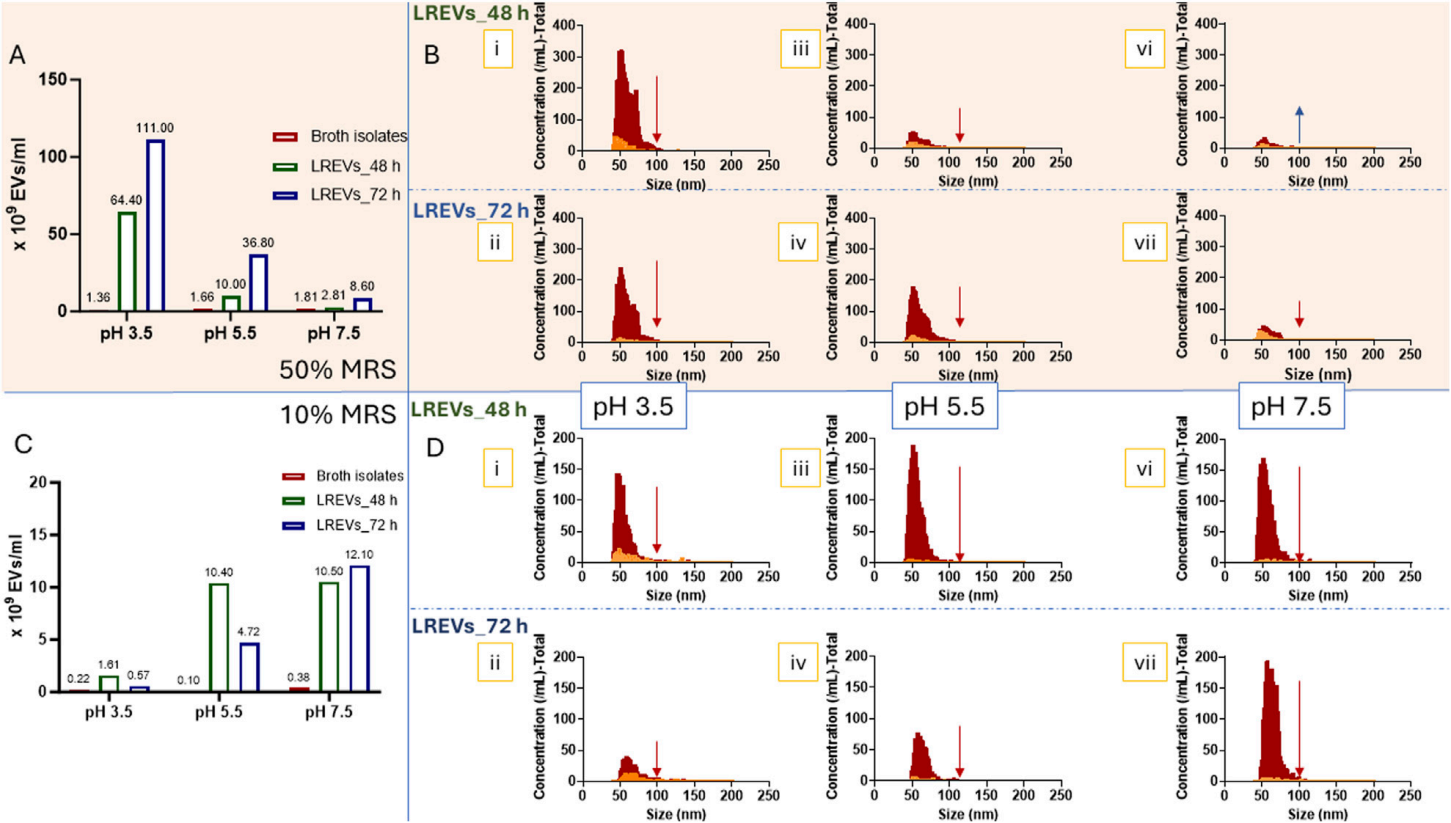
The concentrations of LREVs isolated from *L. rhamnosus* cultured under 50% **(A)** and 10% broth concentrations **(C)**. The size and size distributions of LREVs isolated from *L. rhamnosus* cultured under 50% **(B)** and 10% **(D)** broth concentrations with pH levels of 3.5 (i, ii), 5.5(iii, iv), and 7.5 (vi, viii), and growth/isolation times of 48 h (i, iii, vi) and 72 h (ii, iv, vii). The red size distribution represents the size distribution of LREVs before Triton X-100 treatment, while the yellow size distribution refers to the size distribution after Triton X-100 treatment.

When the broth concentration was reduced to 50%, the number of vesicles found within the broth isolates reduced substantially while a notable increase was observed in the concentration of LREVs particularly under lower pH conditions (pH 3.5) and with prolonged culture times (up to 72 h). The concentration of broth isolates at pH levels 3.5, 5.5, and 7.5 were similarly low. At 48 h, the concentration of LREVs peaked at 6.64 × 10^10^ vesicles/mL at pH 3.5, significantly higher compared to pH 5.5 and pH 7.5, where production was reduced by 85% and 95%, respectively (Figure 4A). Furthermore, after prolonging the culture time to 72 h, the production of LREVs increased 1.7 times for pH 3.5, 3.68 times for pH 5.5, and 3.1 times for pH 7.5, respectively, compared to those isolated at 48 h.

The decrease of broth concentration to 10% further reduced the concentration of broth isolates. However, at this lower broth concentration, the concentrations of LREVs increased with increasing pH (pH 7.5) but these concentrations were subsequently reduced with prolonged culture time. At pH 7.5, the production of LREVs peaked at 1.05 × 10^10^ vesicles/mL at 48 h, while at pH 5.5 and pH 3.5, the production of LREVs was decreased by 5% and 85% respectively compared to pH 7.5 (Figure 4C). Extending the culture time from 48 to 72 h led to a 15% increase in LREV production at pH 7.5. However, at pH 5.5 and 3.5, there were reductions in LREV production by 53% and 65% respectively.

The size and distribution of LREVs isolated from 50% broth were affected by pH but not by growth time. At pH 3.5, LREVs exhibited a bimodal size distribution at 48 h with peaks at 51 nm and 72 nm (Figure 4B i). Prolonging the growth time to 72 h maintained a similar bimodal distribution with peaks at 52 nm and 68 nm (Figure 4B ii). At pH 5.5 and 7.5, LREVs showed near-Gaussian distribution with a mean size of 57 nm. The size distribution remained consistent at both 48 and 72 h (Figure 4B iii-vii).

The size of LREVs isolated from 10% broth was influenced by both pH and growth time. At pH 3.5 and 48 h culture time-point, LREVs displayed a near-Gaussian distribution, with a mean size of 59 nm at (Figure 4D i), which increased to 71 nm at the 72-h culture time-point (Figure 4D ii). Conversely, at pH 5.5, LREVs showed a mean size of 78 nm at 48 h (Figure 4D iii), decreasing to 65 nm at 72 h (Figure 4D iv). LREVs isolated from pH 7.5 had a mean size of 58 nm at 48 h (Figure 4D vi), which also increased to 65 nm at 72 h (Figure 4D vii).

To assess the presence and amount of LREVs obtained from different culture conditions, we exposed LREVs to Triton X-100 and measured the decrease in LREV particle counts. A reduction in particle count within LREVs indicates disruption of LREVs’ membrane integrity by Triton X-100, revealing the number of LREVs within the sample, hence the sample purity.

For LREVs isolated from broth with a 50% concentration, the particle count was pH-, and culture time-dependent, and the most significant drop in the particle count was observed for LREVs obtained in acidic conditions. Specifically, for LREVs isolated at 48 h, there was a 90% and 64% decrease in the particle count at pH 3.5 and 5.5, respectively, after the exposure to Triton X-100. In contrast, a 93% increase in the particle count was observed at pH 7.5 (Figure 4B; Supplementary Table S1). However, extending the culture time to 72 h resulted in the reduction of LREVs secreted in all pH conditions; specifically, the particle count dropped by 96% at pH 3.5, 91% at pH 5.5, and 45% at pH 7.5. These results indicate that, under 50% broth concentration, culturing in acidic culture conditions and prolonged culture time improved LREV purity.

For LREVs isolated from 10% broth concentration, the reduction of particle count was pH-, and culture time-dependent, and the most significant drop in the particle count was shown for LREVs obtained in neutral conditions. Specifically, for LREVs isolated at 48 h under 10% broth concentration, a 79% reduction was observed and for those isolated from pH 3.5 after exposure to Triton X-100, while for both pH 5.5 and pH 7.5, the particle count dropped by 96% (Figure 4D; Supplementary Table S1). Extending growth to 72 h dropped the particle counts within LREVs isolated from pH 3.5, pH 5.5 and pH 7.5 by 62%, 92% and 96%, respectively after treating with Triton X-100. These findings suggest that 10% broth concentration improved LREV purity with increasing pH but decreased with longer culture times.

#### The results of the effect of modified culture conditions on membrane signals of LREVs

To assess the particle-associated lipid content of LREVs obtained from the various culture conditions, we used PKH67 dye labelling. This dye binds specifically to the lipid bilayer of membranes, facilitating visualization and detection of membrane signals of LREV.

Reducing the broth concentration to 50% resulted in a relatively low number of stained vesicles in broth isolates: 20 vesicles at pH 3.5, 27 vesicles at pH 5.5, and 267 vesicles at pH 7.5 (Supplementary Figure S3A). However, at this broth concentration, the number of stained particles within LREVs increased with lowering pH and extended growth time. Specifically, at pH 3.5, 5,738 vesicles were observed in LREVs isolated at 48 h, while this number decreased by 88% at pH 5.5 and by 95% at pH 7.5. However, when the culture time was extended to 72 h, we observed different levels of increase in the number of stained vesicles within LREV isolated from different pHs. Specifically, there was a 2.77-fold increase at pH 3.5, an 8.5-fold increase at pH 5.5, and a 2.3-fold increase at pH 7.5 (Supplementary Figure S3A).

Reducing the broth concentration to 10% resulted in fewer vesicles being stained in broth isolates: 24 vesicles at pH 3.5, 58 vesicles at pH 5.5, and 106 vesicles at pH 7.5 (Supplementary Figure S3C). Under this broth concentration, the number of stained particles within LREV isolates decreased with lower pH and subsequently prolonged growth time. Specifically, at pH 7.5, 5,509 vesicles were observed in LREVs isolated at 48 h, while this number decreased by 42% at pH 5.5 and by 94% at pH 7.5. This number was also affected by prolonged culture time to 72 h, with a 52% decrease at pH 5.5, an 87% decrease at pH 3.5, and a 25% increase at pH 7.5.

The FITC-A signal intensity of LREVs was used to assess the amount of membrane signals within these LREVs. For particles isolated from broth at 50% concertation, FITC-A signal intensity of broth isolates was close to the baseline, while LREVs showed varied signals across different conditions and growth times. Specifically, at 48 h, stained vesicles showed intensities of FITC-A between 10^1^ and 10^3^ for pH 3.5, 5.5, and 7.5 (Supplementary Figure S3B-iii). Extending the growth time to 72 h broadened the signal distribution of FTIC-A to 10^4^ for pH 5.5 and 7.5 (Supplementary Figure S3B-iii). However, prolonging growth times resulted in more stained vesicles with relatively low intensities of FITC-A (10^1^-10^2^) and demonstrated another distinct subpopulation with higher intensities (10^2^-10^3^) (Supplementary Figure S3B). These results suggested that LREVs isolated at 48 h display similar membrane signals regardless of pH conditions, but prolonged growth times to 72 h led to the production of more vesicles with lower membrane signals at pH 3.5, and more vesicles with higher signals at pH 5.5 and 7.5 indicating they have more lipid content.

Under 10% concentration of broth, broth isolates showed baseline-level membrane signals, while LREVs exhibited varying signals across different conditions and growth times. Specifically, at pH 3.5, stained vesicles showed intensities between 10^1^ and 10^3^ of FITC-A at 48 h, diminishing to baseline at 72 h (Supplementary Figure S3D-i). At pH 5.5, intensities of FITC-A ranged from 10^1^ to 10^4^ at 48 h, decreasing to <10^4^ at 72 h (Supplementary Figure S3D.ii). At pH 7.5, intensities of FITC-A ranged from 10^1^ to 10^4^ at 48 h, broadening to >10^4^ at 72 h (Supplementary Figure S3D. iii). These results suggest that LREVs showed increased membrane signals with increasing pH. Prolonged growth times to 72 h decreased signals for pH 3.5 and pH 5.5, while leading to the production of more vesicles with high membrane signals at pH 7.5.

#### The effect of modified culture conditions on biological characteristics of LREVs

In addition to evaluating LREVs’ production and purity, we determined how different culture conditions affect their molecular composition, *i.e.*, protein, carbohydrate, and lipid.

##### The results of effects of pH and growth on protein, carbohydrate and lipid content at 50% broth concentration

At 50% broth concentration, broth isolates had protein contents of 351 μg/mL for pH 3.5, 413 μg/mL for pH 5.5, and 391 μg/mL for pH 7.5 (Supplementary Figure S4A). However, protein concentration in LREVs isolated at 48 h decreased by 14% for pH 3.5, 20% for pH 5.5, and 10% for pH 7.5, compared to those within broth isolates. Extending growth time to 72 h decreased protein concentration by 20% within LREVs from pH 7.5, with no significant difference within LREVs from pH 3.5 and 5.5, compared to those at 48 h. This indicates that the protein composition of LREVs may have been influenced by the protein content of the broth. However, the reduction of protein in LREVs compared to broth isolates could be attributed to protein consumption by *L. rhamnosus* during the culture.

The carbohydrate content of broth isolates showed relatively high concentrations at 50% broth concentration: 117 μg/mL for pH 3.5, 204 μg/mL for pH 5.5, and 204 μg/mL for pH 7.5 (Supplementary Figure S4B). The carbohydrate concentration for/of LREVs isolated at 48 h doubled for pH 3.5 compared to those within broth isolates at same pH but decreased by 59% and 69% for pH 5.5 and pH 7.5, respectively. Extending the growth time from 48 to 72 h had no effect on carbohydrate content. This suggests that at 50% broth, the carbohydrate content with LREVs could be attributed to the broth isolates but may also be influenced by the production of carbohydrates from probiotics at different growth times and pH conditions.

The lipid content within broth isolates was pH-dependent, and at 50% broth concentration, we recorded the following lipid concentrations: 491 μg/mL for pH 3.5, 816 μg/mL for pH 5.5, and 1,101 μg/L for pH 7.5 (Supplementary Figure S4C). The lipid concentration within LREVs isolated at 48 h 100% for pH 3.5 and increased by 32% for pH 5.5, compared to those within broth isolates isolated at same pH but decreased by 13% for pH 7.5, respectively. Extending the growth time from 48 to 72 h did not affect the lipid concentration of LREVs across all pH conditions. This suggested that LREVs isolated from acidic conditions contained a higher amount of lipid compared to those from natural conditions. Prolonged culture time did not affect the lipid content.

#### The results of effects of pH and growth on protein, carbohydrate and lipid content at 10% broth concentration

By reducing broth concentration to 10%, protein content within the broth isolates was reduced to 80 μg/mL for pH 3.5, 54 μg/mL for pH 5.5, and 106 μg/mL for pH 7.5 (Supplementary Figure S4D). The protein concentrations in LREVs at 48 h decreased by 44% for pH 3.5, 46% for pH 5.5, and 94% for pH 7.5, respectively, compared to broth isolates. Extending growth time to 72 h increased protein concentrations with LREVs by 2.1- and 6-fold within LREVs for pH 5.5, and 7.5, respectively, with no significant different difference for pH 3.5 compared to those at 48 h. This suggested that at low broth concentration, protein within the broth was consumed by *L. rhamnosus*, resulting in low protein with LREVs. The protein content within LREVs increased with prolonged growth time likely due to the probiotic secretion.

Reducing broth concentration to 10% also led to a decrease in carbohydrate content within the broth isolates: 43 μg/mL for pH 3.5, 48 μg/mL for pH 5.5, and 56 μg/mL for pH 7.5 (Supplementary Figure S4E). Conversely, carbohydrate concentrations in LREVs isolated at 48 h increased 4 times for pH 7.5, with no significant difference observed for pH 3.5 and pH 5.5, compared to those in broth isolates. Extending growth time to 72 h resulted in a 75% decrease in carbohydrates for LREV isolated from pH 7.5, with no significant effect on LREVs isolated from pH 3.5 and pH 5.5. This suggested that at 10% broth, only LREVs cultured under neutral conditions contained carbohydrates, but carbohydrates decreased with prolonged culture time.

Lipid content within the broth isolates reduced when broth concentration decreased to 10% within 370 μg/mL for pH 3.5, 219 μg/mL for pH 5.5, and 481 μg/mL for pH 7.5 (Supplementary Figure S4F). The lipid concentration within LREVs isolated at 48 h decreased by 63%, 57%, and 68%, respectively, compared to those within broth isolates. Extending the growth time to 72 h increased lipid concentration by 2.1-fold for pH 3.5 and pH 7.5, and 4-fold for pH 5.5 compared to those within LREVs isolate at 48 h. This indicated that, at 10% broth, *L. rhamnosus* used lipids from the broth, reducing lipid content within LREVs. Prolonged growth time increased lipid content within LREVs, suggesting lipid production by *L. rhamnosus* during culture, carried by LREVs.

#### The results of elemental compositions of *L. rhamnosus* and LREVs isolated from modified culture conditions

To investigate whether culture conditions impact on elemental composition of *L. rhamnosus* and the LREVs, we performed an assessment into the elemental composition of LREVs, *L. rhamnosus*, and control broth isolates.

**FIGURE 5:**
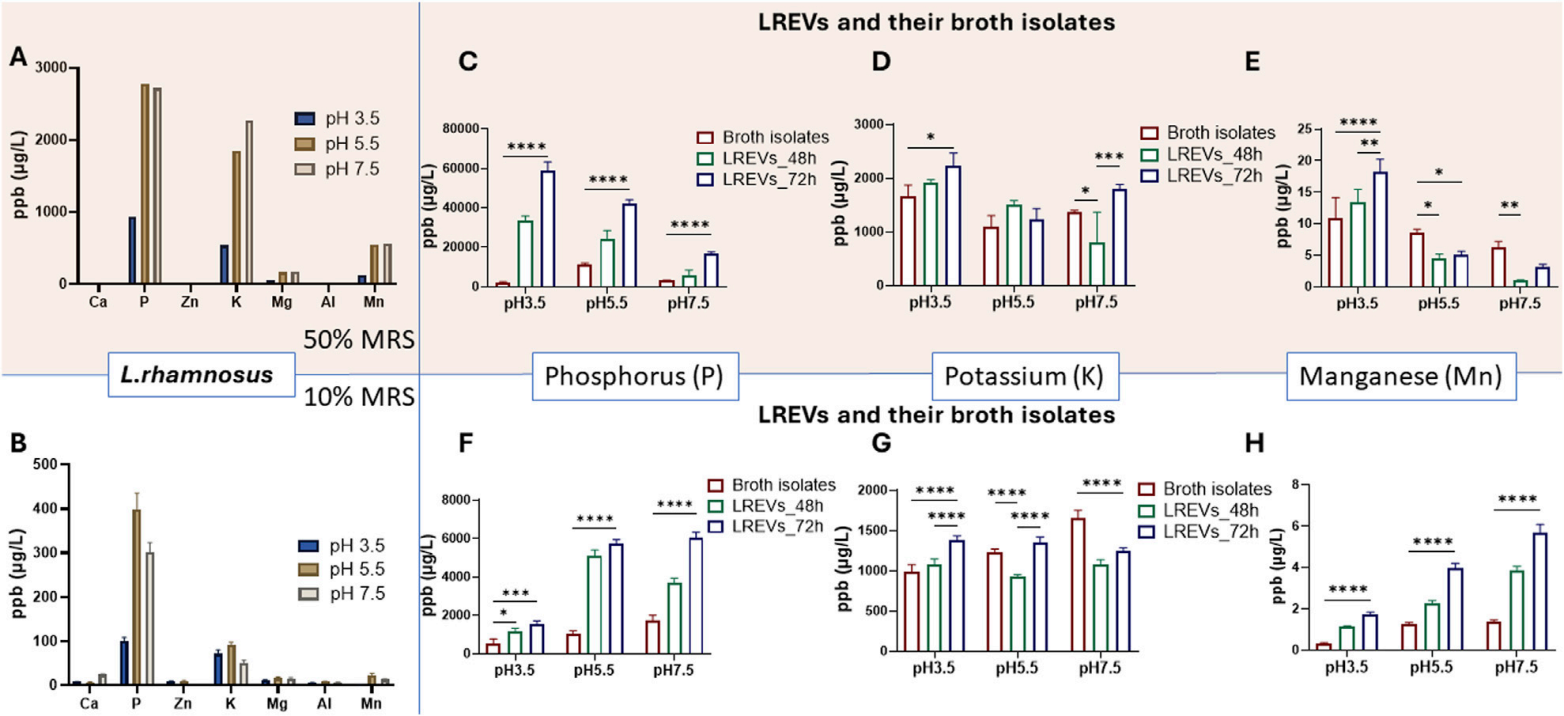
Elemental composition of *L. rhamnosus* culture within 50% of broth **(A)** and 10% of broth concentration **(B)** with different pH conditions. The phosphorus (P) concentrations **(C,F)** potassium (K) concentrations **(D,G)** and Manganese (Mn) concentrations **(E,H)** detected within LREVs isolated at 48 and 72 h as well as broth isolates that were obtained form 50% of broth concentration **(C–E)** and 10% of broth concentration **(F–H)** A *p*-value less than 0.05 was considered statistically significant (ns: *p* > 0.05; *: *p* ≤ 0.05; **: *p* ≤ 0.01; ***: *p* ≤ 0.001; ****: *p* ≤ 0.0001).

The elemental analysis of *L. rhamnosus* showed the presence of phosphorus (P), potassium (K), magnesium (Mg), and manganese (Mn) across different growth conditions, but their relative concentrations varied. For instance, phosphorus and potassium levels were highest at pH 5.5% and 50% broth concentration, while at pH 3.5, these levels decreased substantially (Figure 5A). When broth concentration was reduced to 10%, the concentrations of P, K, Mg and Mn decreased further, especially at pH 3.5 (Figure 5B), indicating a strong association between growth conditions and elemental composition.

Considering the predominance of phosphorus (P), potassium (K), and manganese (Mn) within *L. rhamnosus*, we determined the elemental composition of both LREVs and broth isolates from different culture conditions.

At 50% of broth concentration, relatively low phosphorus (P) concentrations were shown within broth isolates: 2002 ppb at pH 3.5, 11140 ppb at pH 5.5, and 3,194 ppb at pH 7.5 (Figure 5C). The concentration of P within LREVs isolated at 48 h increased significantly, by 16.6-fold for pH 3.5 and 2-fold for pH 5.5, compared to broth isolates, with no significant difference observed for pH 7.5. Extending the culture time to 72 h further increased the P concentration within LREVs, by nearly 2-fold for pH 3.5 and pH 5.5, and 2.9-fold for pH 7.5, compared to those isolated at 48 h. This indicated that the amount of P within LREVs culture under 50% broth increased by the acidity of culture conditions and prolonged culture time.

Reducing broth concentration to 10% led to relatively low P concentrations in broth isolates: 520 ppb at pH 3.5, 1,053 ppb at pH 5.5, and 1765 ppb at pH 7.5 (Figure 5F). However, the concentration of P within LREVs isolated at 48 h increased by 2.2-, 4.8- and 2.1-fold for pH 3.5, pH 5.5 and pH 7.5 compared to those with broth isolates. Prolonging the culture time to 72 h further increased the P concentration within LREVs by 12% and 62% for pH 5.5 and pH 7.5, with no significant difference for pH 7.5. This indicated that the amount of P within LREVs culture at 10% broth increased by neutral of culture conditions and prolonged culture time.

At 50% of broth concentration, broth isolates exhibited K concentrations at similar levels, with 1,664 ppb at pH 3.5, 1,099 ppb at pH 5.5, and 1,364 ppb at pH 7.5 (Figure 5D). The concentration of K within LREVs isolated at 48 h showed a 41% decrease for pH 7.5 compared to broth isolates, with no significant difference observed for pH 3.5 and pH 5.5. For 72 h growth time, the concentration of potassium doubled within LREVs at pH 7.5, there was no change of the K concentration for pH 3.5 and 5.5, compared to those isolated at 48 h. This suggested that the K content within LREVs was influenced by the culture environment, *i.e.*, broth and pH.

When the broth concentration was reduced to 10%, the broth isolates did not affect K concentrations which were: 982 ppb at pH 3.5, 1,235 ppb at pH 5.5, and 1,655 ppb at pH 7.5 (Figure 5G). The K concentration was lower in LREVs isolated at 48 h when compared with broth isolates. Specifically, it decreased by 25% for pH 5.5% and 34% for pH 7.5, with no significant difference observed for pH 3.5. After 72 h of culture, K concentration within LREVs increased by 28%, 45% and 15% for pH 3.5, pH 5.5 and pH 7.5respectively compared to those within LREVs isolated at 48 h. This suggested that the K content within LREVs was influenced by the K present in the broth isolates, with this influence varying depending on the pH conditions.

At 50% of broth concentration, broth isolates exhibited relatively higher Mn levels, with concentrations of 10.8 ppb at pH 3.5, 8.6 ppb at pH 5.5, and 6.3 ppb at pH 7.5 (Figure 5E). The concentration of Mn within LREVs isolated at 48 h decreased by 47% for pH 5.5 and by 85% for pH 7.5, compared to broth isolates, with no significant difference for pH 3.5. Prolonged the culture time to 72 h increased the Mn concentration within LREVs by 36% for pH 3.5, with no significant difference for pH 5.5 and pH 7.5 compared to those isolated at 48 h. This suggested that broth contained high amounts of Mn, with a higher chance of transfer to LREVs under acidic conditions.

At 10% of broth concentrations, the Mn concentrations within broth isolates reduced to 0.3 ppb at pH 3.5, 1.3 ppb at pH 5.5, and 1.4 ppb at pH 7.5 (Figure 5H). For LREVs isolated at 48 h, increases in the Mn concentration were observed for all conditions, compared to broth isolates, by 3.6-,1.7- and 2.7-fold for pH 3.5, pH 5.5, and pH 7.5, respectively. At 72 h of culture, Mn concentrations within LREVs increased by 55%, 76% and 49% for pH 3.5, pH 5.5, and pH 7.5 respectively, compared to those isolated at 48 h. These findings indicated that at 10% broth concentration, Mn carried by LREVs increased under neutral conditions and with prolonged culture times.

### The results of effects of LREVs isolated from different culture conditions on skin cell viability

A cell viability (metabolic activity) study was conducted on human epidermal keratinocytes (HEK) to assess the safety of LREVs isolated under various conditions, with broth isolates as controls. The impact of LREVs on cells was tested using two EV concentrations: 10^2^ EVs per cell and 10^4^ EVs per cell. Broth isolates were used as the control experiments. Since the broth isolates contain negligible number of particles, to ensure the same treatment conditions we used the broth isolates at the same volume/dilution ratios as for LREVs.

LREVs and broth isolates, whether obtained from 50% or 10% broth concentration and regardless of isolation time and pH conditions, exhibited minimal effect on cell viability at a low EV concentration (10^2^ EVs per cell) compared to untreated controls (Supplementary Figures S5A, B). However, upon increasing the treatment concentration to 10^4^ EVs per cell, some broth isolates exhibited toxicity on cells when treated at the same volume as LREVs. The toxicity from broth could impact LREVs isolated from same conditions, causing them to demonstrate toxicity. Nonetheless, when treated at equivalent volume/dilution ratios, LREVs consistently showed lower toxicity compared to broth isolates.

**FIGURE 6:**
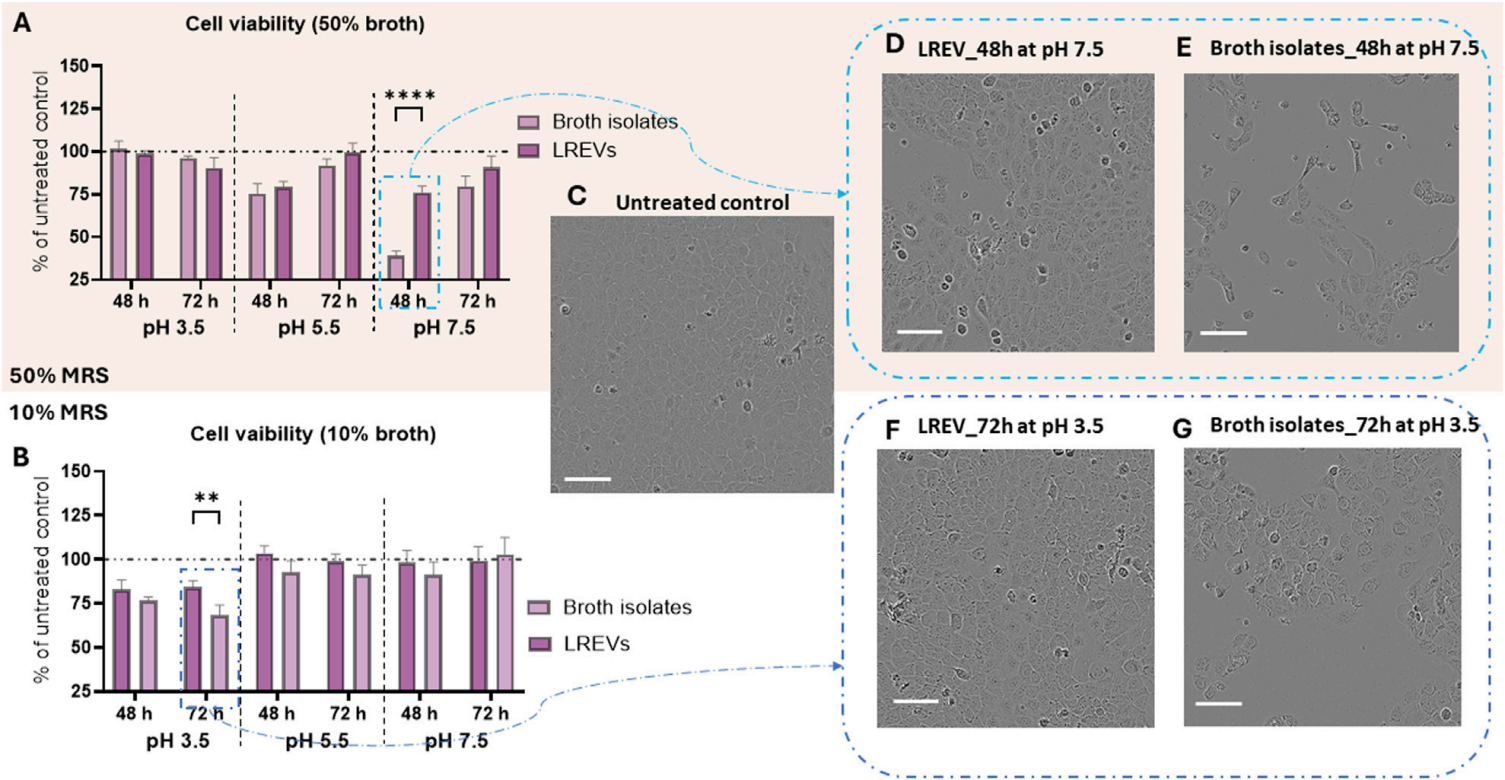
Cell viability of human epidermal keratinocyte (HEK) that treated by LREVs at concentration of 10^4^ EVs per cells and broth isolates at the same volume/dilution ratio as LREVs including cell viability of HEK treated by LREVs and broth isolates that isolated from 50% of broth concentration **(A)** and 10% of broth concentration **(B)**. Cell morphology and density of untreated cell control **(C)**, cell treated with LREV that isolated at 48 h from 50% of broth concentrations and pH 7.5 condition with a concentration of 10^4^ EVs per cell **(D)**, and cell treated with broth isolates from similar condition with same treatment volume **(E)**, as well as cell treated with LREV that isolated at 72 h from 10% of broth concentrations and pH 3.5 condition with a concentration of 10^4^ EVs per cell **(F)**, and cell treated with broth isolates from similar condition with same treatment volume **(G)** (Scale bar: 100 µm). A *p*-value less than 0.05 was considered statistically significant (ns: *p* > 0.05; *: *p* ≤ 0.05; **: *p* ≤ 0.01; ***: *p* ≤ 0.001; ****: *p* ≤ 0.0001).

For instance, when cells were treated with LREVs isolated under pH 5.5 at 48 h (50% broth concentration) and those treated with broth isolates at the same volume as LREVs, they exhibited 80% viability (Figure 6A). When comparing the effects on cell viability between LREVs isolated under pH 7.5 at 48 h (50% broth concentration) and broth isolates used at the same volume as LREVs, differences emerged. While cell viability decreased to 40% when treated with broth isolates, it significantly increased to 76% when treated with LREVs.

Similarly, when comparing cell viabilities treated by LREVs isolated under pH 3.5 at 48 h (10% broth concentration) and those treated by broth isolates at the same volume as LREVs, 80% of cell viability was observed in both treatments (Figure 6B). However, when comparing cell viability of cells when treated with LREVs isolated under pH 3.5 at 72 h (10% broth concentration) and those treated by broth isolates at the same volume as LREVs, the cell viability treated with broth isolates dropped to 68%, whereas for the cells treated by LREVs, cell viability significantly increased to 75%.

The effects of LREVs and broth isolates on cell viability were also demonstrated through evaluation of cell density and morphology. LREVs isolated from 50% to 10% broth concentrations, had similar cell density as nontreated control (Figures 6D, F) when compared with untreated cells (Figure 6C). This means LREVs produced in this culture conditions are non-toxic. However, their corresponding broth isolates reduced cell density and appearance of the “stressed” morphology, characterized by morphological change with cell elongation (Figures 6E, G). These findings suggest that cells treated with LREVs exhibited better health compared to those treated with broth isolates, as evidenced by their enhanced cell density, and improved morphology.

### The results of effects of LREVs isolated from different culture condition on antimicrobial activity

To determine whether LREVs and broth isolates have antimicrobial activity, we conducted minimum inhibitory concentration (MIC) tests using *S. aureus* bacteria strains. LREVs and broth isolates were diluted to working concentrations ranging from 5% to 0.625% (v/v) on *S. aureus*.

**FIGURE 7:**
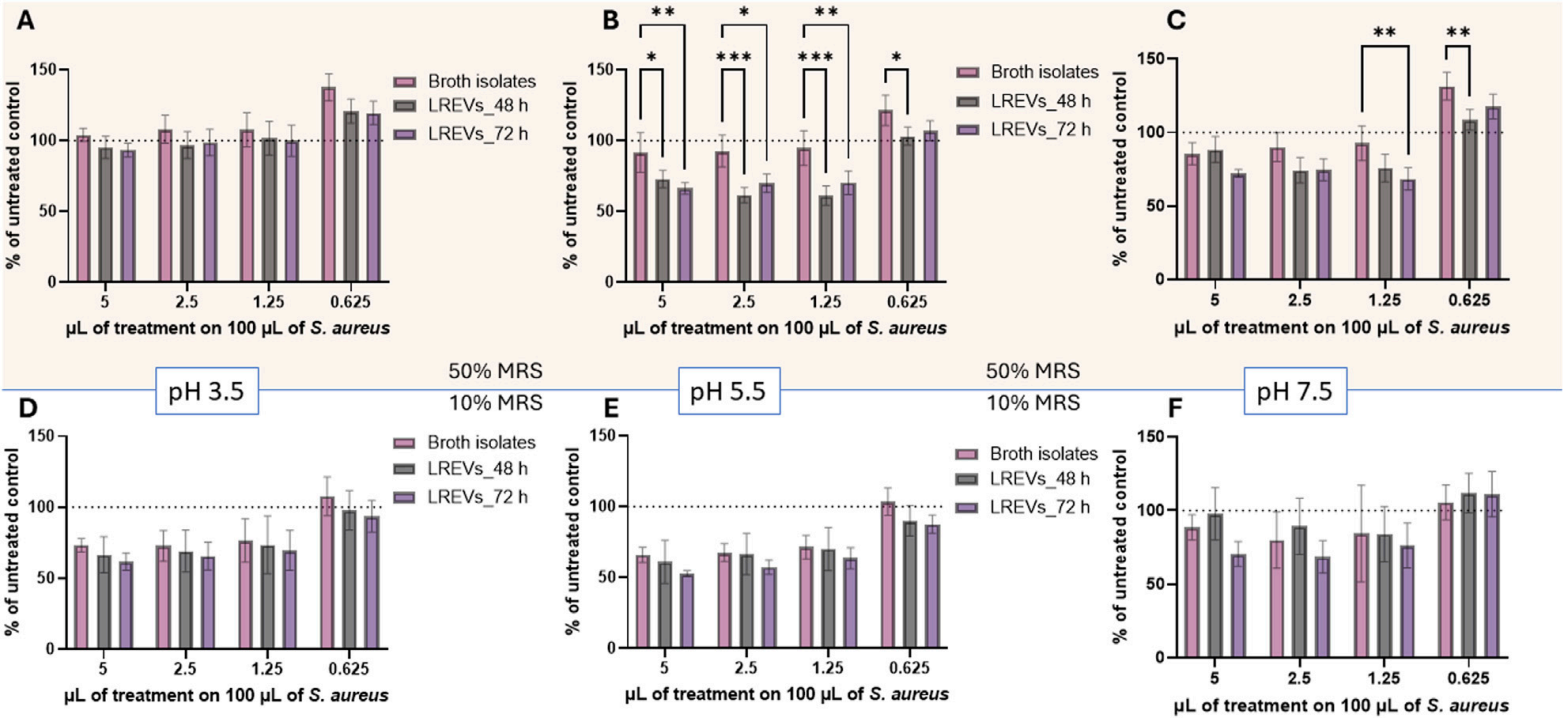
Percentage of growth of *s. aureus* strains incubated with LREVs and broth isolates across concentration range from 5% to 0.625% (v/v), with the optical density OD_600_ of untreated *S. aureus* set as 100%. The results include **(A–C)** percentage of growth of *S. aureus* strains for LREVs isolated at 48 and 72 h, as well as their respective broth isolate, isolated at 50% broth concentration with pH values of 3.5, 5.5, and 7.5, respectively. **(D–F)** percentage of growth of *S. aureus* strains for LREVs isolated at 48 and 72 h, as well as their respective broth isolate, isolated at 10% broth concentration with pH values of 3.5, 5.5, and 7.5, respectively. A *p*-value less than 0.05 was considered statistically significant (ns: *p* > 0.05; *: *p* ≤ 0.05; **: *p* ≤ 0.01; ***: *p* ≤ 0.001).

LREVs produced at pH 3.5% and 50% broth concentration showed no inhibition of *S. aureus* growth regardless of the concentration (Figure 7A). At the lowest concentration (0.625%) there was a 20% increase in *S. aureus* growth for LREVs isolated at 48 and 72 h, and a 40% increase for broth isolates. There were no statistically significant differences between the MIC between LREVs and broth isolates. This suggests that LREVs obtained at pH 3.5% and 50% broth concentration lack antimicrobial properties against *S. aureus* and have effects comparable to broth isolates.

In contrast, LREVs produced at pH 5.5 and in 50% broth concentration inhibited bacterial growth at concentrations of 5%, 2.5%, and 1.25%. No inhibition was observed for broth isolates at these dilutions (Figure 7B). The growth of *S. aureus* reduced by 28% (for 5%) and 39% (for 2.5% and 1.25%) for LREVs isolated at 48 h, and 34% (for 5%) and 30% (for 2.5% and 1.25%) for those isolated at 72 h. No inhibition of the bacterial growth was observed at a concentration of 0.625%. There were no significant differences observed in the bacteria growth inhibition between the effects of LREVs isolated at 48 h and those isolated at 72 h. This study showed that LREVs produced at pH 5.5 have antimicrobial activity against *S. aureus* strains–this activity was not observed for broth isolates.

LREVs produced at pH 7.5% and 50% broth concentration inhibited bacterial growth at dilutions of 5%, 2.5%, and 1.25% (Figure 7C). The growth inhibition was 15% (for 5%) and 10% (for 2.5%) and was the same for both probiotic culture times: 48 and 72 h. The bacterial growth inhibition was also observed for broth isolates and there were no statistical differences between LREVs and broth isolates. Interestingly, at a concentration of 1.25%, a 30% bacterial growth inhibition was observed for LREVs isolated at 72 h. For higher dilutions, none of the samples affected bacterial growth. These results showed that LREVs produced at pH 7.5 have a concentration-dependent antimicrobial activity against *S. aureus* strains, which could be associated with co-isolated components of the broth, which also showed some antimicrobial activity.

LREVs produced in 10% broth and at pH 3.5, pH 5.5 and pH 7.5 showed antimicrobial activity against *S. aureus*. However, the activity was at the same level as observed for broth isolates (Figures 7D–F). The bacterial growth was reduced by 23%–34% (for dilution 5%, 2.5% and 1.25%), for LREVs produced at pH 3.5 isolated at 48 h. While for LREVs produced at pH 5.5 (48 h), the growth reduction was 29%–39% and for LREVs produced at pH 7.5 (48 h) was 3%–15%. No growth reduction was observed at the lowest concentration. Statistical analysis revealed no significant differences in antimicrobial activity between LREVs isolated at 48 h and those isolated at 72 h, across all dilutions. This was also true for the corresponding broth isolates. This means that the antimicrobial activity of LREVs produced under these culture conditions might be attributed to the co-isolated components of the broth.

## Discussion

Here we systematically studied the effect of environmental cues, *i.e.*, pH and broth concentrations on the production, purity and functionality of probiotic EVs. Full-strength broth (100% MRS broth concentration) is typically used for probiotic culture, and it constitutes a microenvironment in which EVs from probiotics are secreted (Croatti et al., 2022; Lee B.-H. et al., 2023). Like serum in mammalian cell culture, culture broth in bacterial culture serves as a source of nutrients and provides an environment for bacteria growth. However, some studies indicated that the use of serum, *i.e*., foetal bovine serum (FBS) in mammalian cell culture, comprising both vesicular and non-vesicular particles, influences the production of mammalian EVs (Lehrich et al., 2021). Additionally, other studies showed that proteins and lipoproteins in serum have similar sizes as EVs, resulting in the co-isolation of serum components when using size/density-dependent EV isolation methods (German et al., 2006; Sun et al., 2019; Brennan et al., 2020). These findings led to the hypothesis that using a full-strength broth could introduce undesirable broth components, considered as contaminates, during the probiotic EV preparations. To test this hypothesis, we first examined the size and concentration of broth components isolated from full-strength broth. We found that MRS broth-derived particles and EVs isolated from *L. rhamnosus* (LREVs) cultured in the same broth had similar sizes. Moreover, when treated with Triton X-100, a reagent that disrupts lipid membrane and reduces the EV numbers (Deville et al., 2021), we observed an increase in the number of both LREVs and broth isolates. This study was in agreement with lipid staining experiments that showed low lipid staining signals in both broth isolates and LREVs (Figure 2), suggesting a low lipid membrane content in LREVs and broth isolates from full-strength broth. No differences in protein, carbohydrate, and lipid concentration, as well as in elemental composition, were observed between LREVs and broth isolates, indicating that they had similar biochemical composition. Collectively, we conclude that broth-derived particles, considered contaminants, were co-isolated with EVs, dominated the sample composition and hindered the accurate identification of LREVs. However, most of the previous studies investigating the physical characteristics and functions of probiotic EVs did not consider the impact of broth contamination and its contribution to the isolated samples (Kurata et al., 2022; Fan et al., 2024; Yang et al., 2024), which might explain some inconsistencies in the results. We recommend eliminating broth contaminants from EV preparations to ensure that the observed effects are solely due to LREVs and not confounded by the broth co-isolates.

It is well-established that microenvironmental conditions affect cell growth, which leads to changes in the secretion of EVs (Tao and Guo, 2020). Hence, we further hypothesised that the broth concentration and environmental pH affect the secretion of LREVs and their physicochemical and biological properties. We determined whether the modification of the nutrient level, specifically broth concentration, could improve the production of probiotic EVs. Our studies showed that changing the broth concentration to 50% and further to 10% reduced the broth-derived contaminants by 30% and 95%, respectively. This is consistent with studies on the production of EVs from mammalian cells, which showed that reducing serum levels using serum-free medium or serum replacement can eliminate serum-derived contaminations in EV preparations (Forteza-Genestra et al., 2020).

As previously reported, changes to the culture conditions, including nutrient levels, can cause stress to bacteria (Dawan and Ahn, 2022). This, in turn, can be used as a powerful strategy to control EV production. The use of environmental stressors (*i.e.*, glucose starvation) was previously explored for increasing the release of EVs from cardiomyocytes and neuronal cells (Garcia et al., 2015). In our study, we found that reducing broth concentrations to 50% (pH 5.5) resulted in a ∼8-fold increase in LREV production, indicating that nutrient starvation improves the production of probiotic EVs. Our study also confirmed that the EVs from this condition maintained a consistent size distribution for 4 weeks (Supplementary Figure S6A), indicating no aggregation or degradation. However, the zeta potential shifted by 5 mV towards less negative values (Supplementary Figure S6B), suggesting changes to the surface properties and the need for alternative storage conditions or buffers to maintain these properties. Taken together, our study demonstrated that reducing broth concentration had dual benefits: (i) effectively reduced broth contaminants, and (ii) increased probiotic EV production.

In addition to nutrient levels, pH can also be utilised to modulate EV production, as demonstrated by previous studies on mammalian cells that showed that low pH affects EV secretion (Nakase et al., 2021). We observed that the low pH of cell culture media, *i.e*., pH 3.5% and 50% broth concentration, induced the highest LREV production. The EV concentration increased ∼3-fold when compared with those produced at pH 5.5% and 50% of broth concentration. However, the studies on mammalian cells and gram-negative bacteria, *Helicobacter pylori,* showed that low pH reduces the production of mammalian EVs and outer membrane vesicles (OMVs), which is inconstant with our finding (Nakase et al., 2021; Johnston et al., 2023). We speculated that LREVs possess different membrane properties inherited from *L. rhamnosus* enhancing their integrity in acidic conditions, supported by studies demonstrating the resilience of *Lactobacillus* strains in maintaining their morphology and integrity in acidic environments (Corcoran et al., 2005). Therefore, we conclude that using acidic pH could promote the release of probiotic EVs and these EVs exhibit resistance to acidic environments.

Nutrient level and pH are considered two types of microenvironmental stressors. However, one can assume that when they are combined, they may have some additive or synergistic stress effects. This means that modifying concentration and pH simultaneously may induce a higher (or different) level of stress on *L. rhamnosus*, consequently affecting LREV production. The initial stress levels were defined by differences in growth rates under various culture conditions (Supplementary Figure S2) with a ‘low’ growth rate indicating a ‘high’ stress level. Moreover, when bacteria reached the stationary phase, the prolonged culture time suggests that cells experienced more cellular stress from stressors like nutrient limitations or pH changes (Navarro Llorens et al., 2010). In summary, Table 2 presents the stress levels for each condition, based on growth rate and stationary phase duration. We found that when stress level <4, the higher stress levels had greater EV production. This finding aligns with studies on the release of OMVs from gram-negative bacteria, where increased vesiculation occurs as a stress response mechanism to enhance bacterial survival under stress conditions (McBroom and Kuehn, 2007). However, the excessively high-stress levels (level 4 and level 5), particularly at 10% broth concentration and pH 3.5, resulted in a decline in LREV production. This could be attributed to acidic pH (pH 3.5) and starvation (10% broth) causing bacterial death and consequently the reduction in viable EV-producing bacteria (Mohiuddin et al., 2021) and degradation of EVs (Cheng Y. et al., 2019). This indicated that the stress levels induced by pH, broth concentration and growth times can be used to control probiotic EV production.

**TABLE 2:** Stress levels of *L. rhamnosus* cultured under various conditions.

Broth concentrations		50%			10%		
pH		3.5	5.5	7.5	3.5	5.5	7.5
Culture time	48 h	2	N/A	N/A	4	3	3
	72 h (+1)	3	1	1	5	4	4
		Initial stress levels: N/A (OD600 0.7-0.9); level 1 (OD600 0.5 -0.7); level 2 (OD600 0.3 -0.5); level 3 (OD600 0.1 -0.3); level 4 (OD600 0.01 -0.1)					

In addition to EV production, our work showed that the protein, carbohydrate and lipid content within LREVs was affected by the culture conditions. We demonstrated that LREVs contain lipids, proteins, and carbohydrates, consistent with previous reports on cargoes of bacterial EVs (Yu et al., 2018). Moreover, we showed that reducing broth concentration decreased protein, carbohydrate, and lipid content in LREVs. However, under certain conditions, broth still contained higher levels of lipid, protein and carbohydrate compared to those within LREVs, indicating that LREVs retain biological components from the broth. For example, we observed increased protein, carbohydrate, and lipid levels in LREV isolated from pH 5.5 and 7.5, when the culture time was increased from 48 to 72 h. The increase could result from the increase in cell stress by prolonged culture causing EVs to carry more specific stress-response cargoes like protein and DNA (Wan et al., 2022). Such increases were not observed within LREVs isolated from pH 3.5, probably due to the acid-induced breakdown. Overall, we conclude that the broth concentration and pH of the culture media, both considered as microenvironmental stressors, influence the composition of LREVs. Our findings are consistent with previous reports that showed that temperature-induced stress altered the molecular composition of EVs produced by fibroblasts (Mohammadipoor et al., 2023).

It is well-established that probiotic haemostasis involves the regulation of metal ions, including transition metals like iron, zinc, and manganese, as an array of nutrients for development, growth, and metabolic activities (Pajarillo et al., 2021). Our studies, for the first time, determined the elemental composition of *L. rhamnosus* and secreted LREVs under different culture conditions. We found that *L. rhamnosus* cultured in 100% broth concentration contained phosphorus (P), potassium (K), magnesium (Mg), and manganese (Mn). These elements remained as domain compositions within *L. rhamnosus*, even as broth concentration decreased to 50% and 10%.

We showed that probiotic EVs contain phosphorus (P), potassium (K), and manganese (Mn), with P and K as dominant elements and Mn present in smaller amounts. We found that concentrations of K within LREVs were not affected by the culture conditions, likely due to the influence of storage buffer. However, P and Mn demonstrated more significant differences, possibly related to the conditions of *L. rhamnosus* and LREV production. Our studies showed an increase in EV concentration led to the increased amount of P. This could be explained by the fact that EVs contain substantial amounts of phospholipids (Yang et al., 2024) and aligns with the role of P in forming fundamental cellular structures, including nucleic acids and membrane phospholipids (Kolodiazhnyi, 2021). In contrast, broth isolates contained relatively low phosphorus concentrations, indicating that the co-isolated broth lipids were not phosphate-based, such as polysorbate. We also observed that the amount of Mn with LREVs increased when stress levels of *L. rhamnosus* increased (Table 2). This aligns with the protective role of Mn against oxidative damage during bacterial growth (Poddar et al., 2021), suggesting that LREVs could carry more Mn, possibly involving proteins such as Mn-specific enzymes for protection during oxidative stress (Martin and Waters, 2022). Collectively, we concluded that P and Mn were key elements within *L. rhamnosus* and were selectively packed into LREV production. This selective behaviour aligns with studies of the mechanism of EVs, selectively sorting and packaging various molecule (Lee et al., 2024).

While the EV concentration, structure and composition are their key quality attributes, the biological functionality of EVs defines their utility for various applications. Here we assessed the safety of LREVs on human keratinocytes and their antimicrobial effects against *S. aureus*. Our studies showed LREVs were safe at low concentrations (10^2^ vesicles per cell) when tested using human keratinocytes. At high concentrations (10^4^ vesicles per cell), LREVs from pH 7.5% and 50% broth and pH 3.5% and 10% broth isolated at 48 h inhibited keratinocyte growth. This inhibitory effect was similar to broth isolates obtained under the same conditions, indicating that the reduction in metabolic activity and cell growth was likely due to broth contaminants rather than LREV properties. Lee *at al.* previously reported that EVs from *Lactobacillus paracasei* were safe when tested with human keratinocytes (Lee K.-S. et al., 2023), which is consistent with our findings.

Since EVs derived from *Lactobacilli* have shown potential in combating infections, including those caused by *S. aureus* (Mata Forsberg et al., 2019), we conducted minimum inhibitory concentration tests with LREVs on *S. aureus.* Antimicrobial activity was only confirmed for LREVs produced at pH 5.5 and pH 7.5 in 50% broth. The antimicrobial activity of EVs produced under these conditions may be linked to antimicrobial compounds such as organic acids (e.g., lactic acid and citric acid) and bacteriocins produced *by L. rhamnosus* (Tkhruni et al., 2020; Ibrahim et al., 2021; Szczerbiec et al., 2022). It is worth noting that LREVs, which showed antimicrobial activity, were not produced under culture conditions that enabled the highest yield of EV production (which was achieved at pH 3.5% and 50% broth). This means that the maximum yield of EV production does not guarantee the desired therapeutic efficacy of EVs. Thus, the yield should not be used as a sole parameter to optimise culture conditions. When designing EV production conditions, it is necessary to define their application and then guide the process to ensure EVs maintain their integrity and possess specific biological activity to achieve the desired therapeutic efficacy.

## Conclusion

In conclusion, culture broth contains biomolecules and components that are typically co-isolated with probiotic EVs, potentially overshadowing the presence of probiotic EVs when full-strength broth is used. However, reducing the broth concentration can effectively eliminate broth-derived contaminants within probiotic EVs. By optimizing stress factors such as culture pH, broth concentrations, and growth time, we can obtain LREV with high production and purity while modulating their biological and elemental composition. Notably, LREVs exhibit no toxicity to skin cells when the impact of broth is managed, and they demonstrate unique antimicrobial effectiveness against *S. aureus* when isolated under optimal conditions. However, achieving the highest LREV production and purity does not necessarily guarantee therapeutic effectiveness, indicating the necessity to maintain LREV’s integrity and preserve their biological activity to achieve therapeutic efficacy.

However, other environmental factors need to be considered in further investigations, including temperature, and the concentrations of individual compounds such as proteins, glucose, salts, and oxygen levels, as these factors could significantly affect probiotic EV production and functionality. In addition to culture conditions, parameters related to the isolation process, such as different isolation methods and post-isolation processing, are critical for ensuring the desired purity and functionality of EVs. Additionally, the different components of probiotic EVs, such as specific phospholipids and proteins, and their contributions to EV functionality has not been fully resolved. Moreover, the stability of LREVs various culture conditions needs additional testing to ensure their properties and functionality for various applications.

Overall, our study has revealed, for the first time, that controlling the environmental stressors including broth concentration, environmental pH and growth time could affect the production and composition of probiotic EVs. Our studies provided new understanding of probiotic responses to environmental stressors and lay the foundation for innovative probiotic EV-based therapies. By exploiting this knowledge, customized probiotic-derived EVs can be designed for various applications. Of note is that the design of EVs must consider the context of use to enable their full potential.

## Objective

To establish precision biomanufacturing methods to produce customised probiotic-derived EVs tailored to the specific characteristics of medical and industrial applications. This will be achieved by advancing our understanding of the role of the microenvironment, *i.e.*, culture conditions, on the production and functionality of EVs. These methods will enable efficient, high-yield, and cost-effective production of EVs and address limitations in the existing culture method. Importantly, these methods will enable the production of bespoke EVs that consider the context of use.

## Data Availability

The original contributions presented in the study are included in the article/Supplementary Material, further inquiries can be directed to the corresponding author.
