# Supplementary material for: Bioprocessing strategies for enhanced probiotic extracellular vesicle production: culture condition modulation
Source: Front Bioeng Biotechnol. 2024 Aug 30;12:1441552. doi: 10.3389/fbioe.2024.1441552 (PMC11392866; doi:10.3389/fbioe.2024.1441552)
Supplement: Supplementary file 1 [file DataSheet1.docx]

Supplementary Material

**Size 1**

**Size 2**

**Size 3**

**Supplementary Figure 1**. The size and size distribution of broth isolates and LREVs that isolated from conventional culture method.


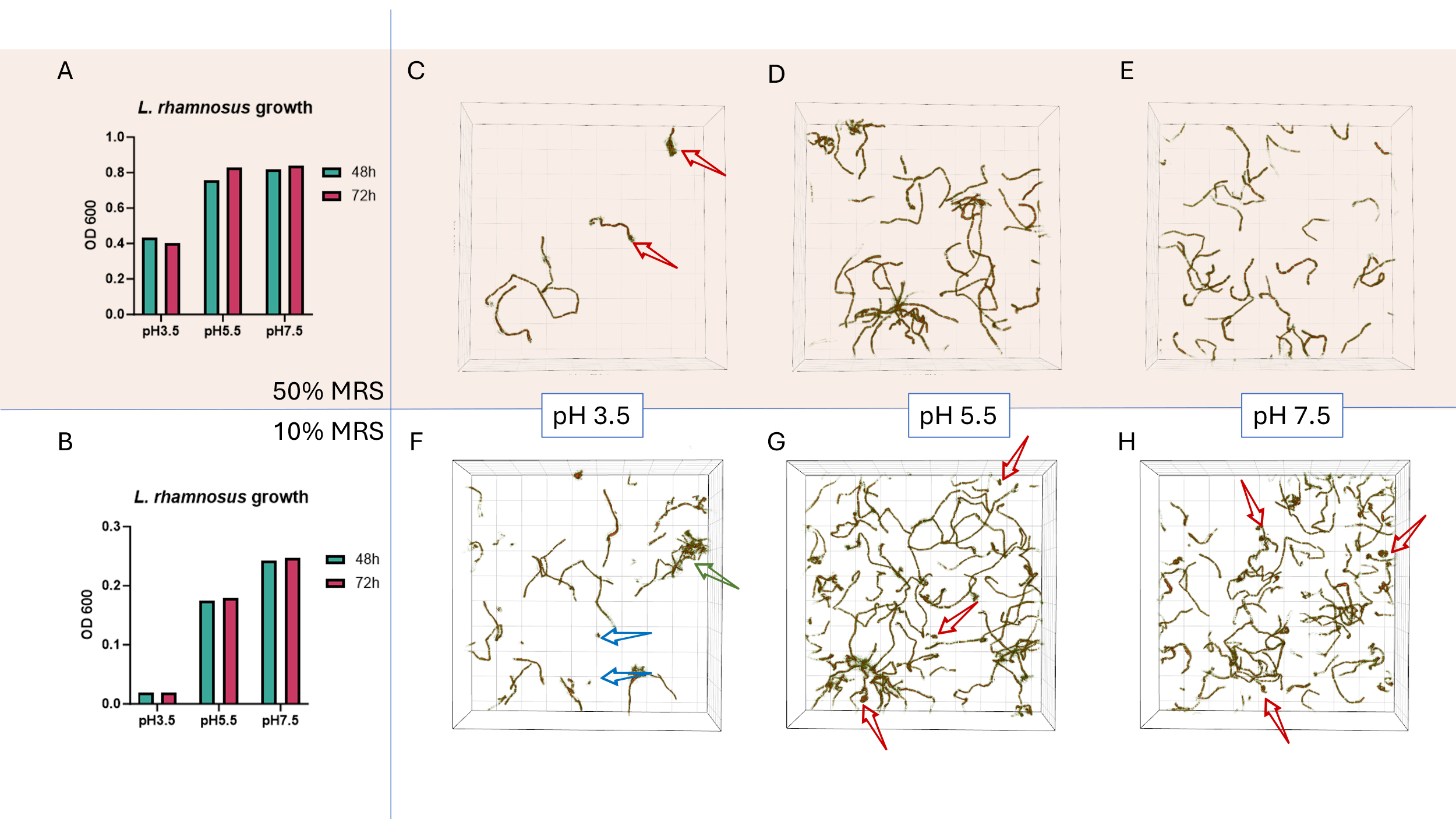


**Supplementary Figure 2.** Growth and morphology of *L.rhamnosus* cultured in modulated conditions. **(A)** the OD_600_ of *L.rhamnosus* grown in pH 3.5, pH 5.5 and pH 7.5 under 50% broth concentration at 2 time points. **(B)**. the OD_600_ of *L.rhamnosus* grown in pH 3.5, pH 5.5 and pH 7.5 under 10% broth concentration at 2 time points. **(C-E)**. the morphology of *L.rhamnosus* grown in pH 3.5, pH 5.5 and pH 7.5 under 50% broth concentration imaged using Nanolive imaging. **(F-G)**. the morphology of *L. rhamnosus* grown in pH 3.5, pH 5.5 and pH 7.5 under 10% broth concentration using Nanolive imaging.


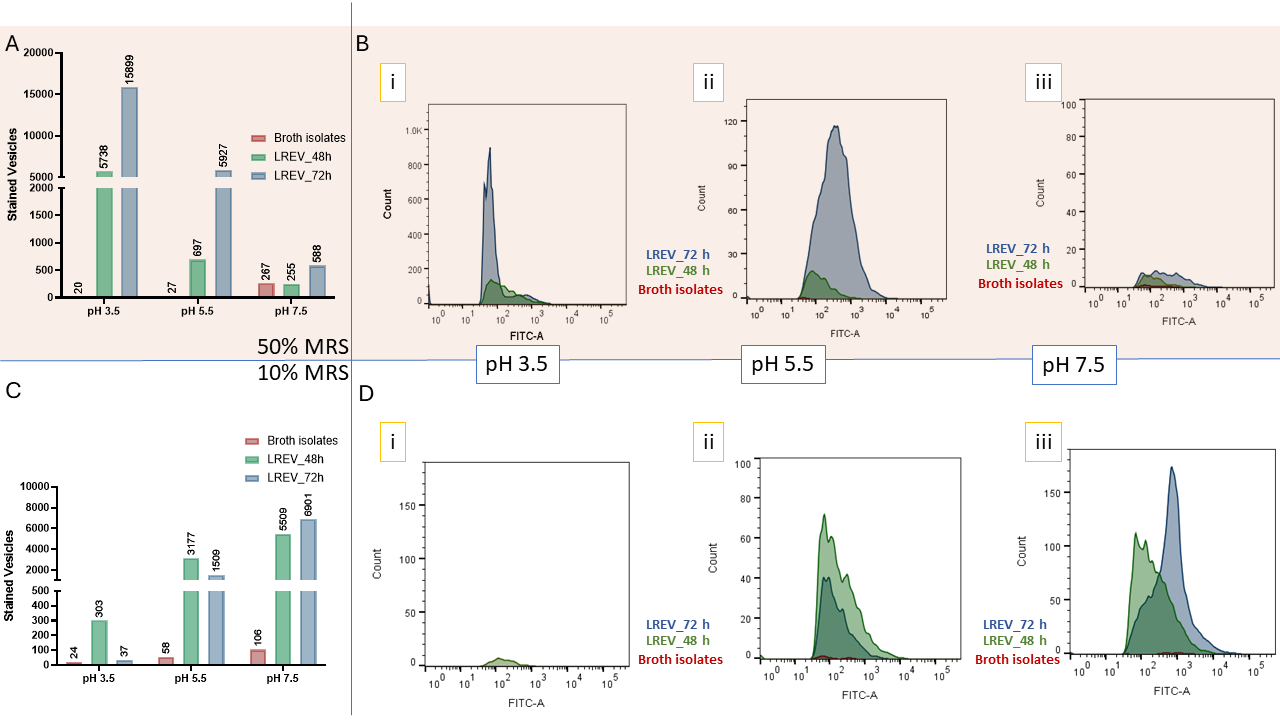
 **Supplementary Figure 3.** The number of stained vesicles **(A, C)** and their distribution **(B, D)** within broth isolates and LREVs that isolated from *L. rhamnosus* cultured under 50% and 10% broth concentrations with pH levels of 3.5, 5.5, and 7.5, and growth/isolation times of 48 h and 72 h.


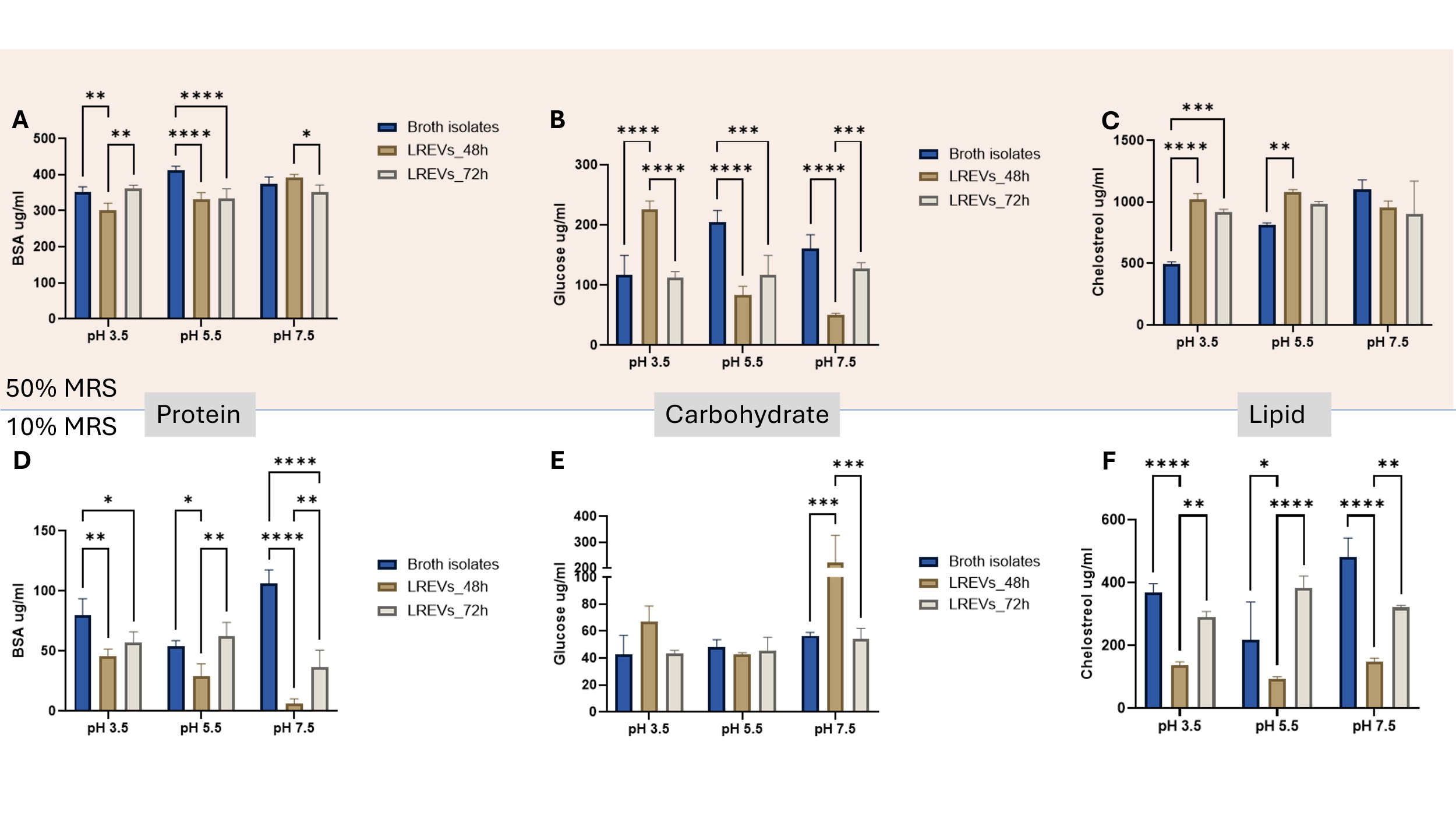


**Supplementary Figure 4.** The protein **(A)**, carbohydrate **(B)** and lipid **(C)** concentrations detected within broth isolates as well as LREVs isolated at two growth time (48 h and 72 h) and three pH conditions (pH 3.5, pH 5.5 and pH 7.5) at 50% of broth concentration. The protein **(D)**, carbohydrate **(E)** and lipid **(F)** concentrations detected within broth isolates as well as LREVs isolated at two growth time (48 h and 72 h) and three pH conditions (pH 3.5, pH 5.5 and pH 7.5) at 10% of broth concentration. A p-value less than 0.05 was considered statistically significant (ns: p > 0.05; *: p ≤ 0.05; **: p ≤ 0.01; ***: p ≤ 0.001; ****: p ≤ 0.0001)


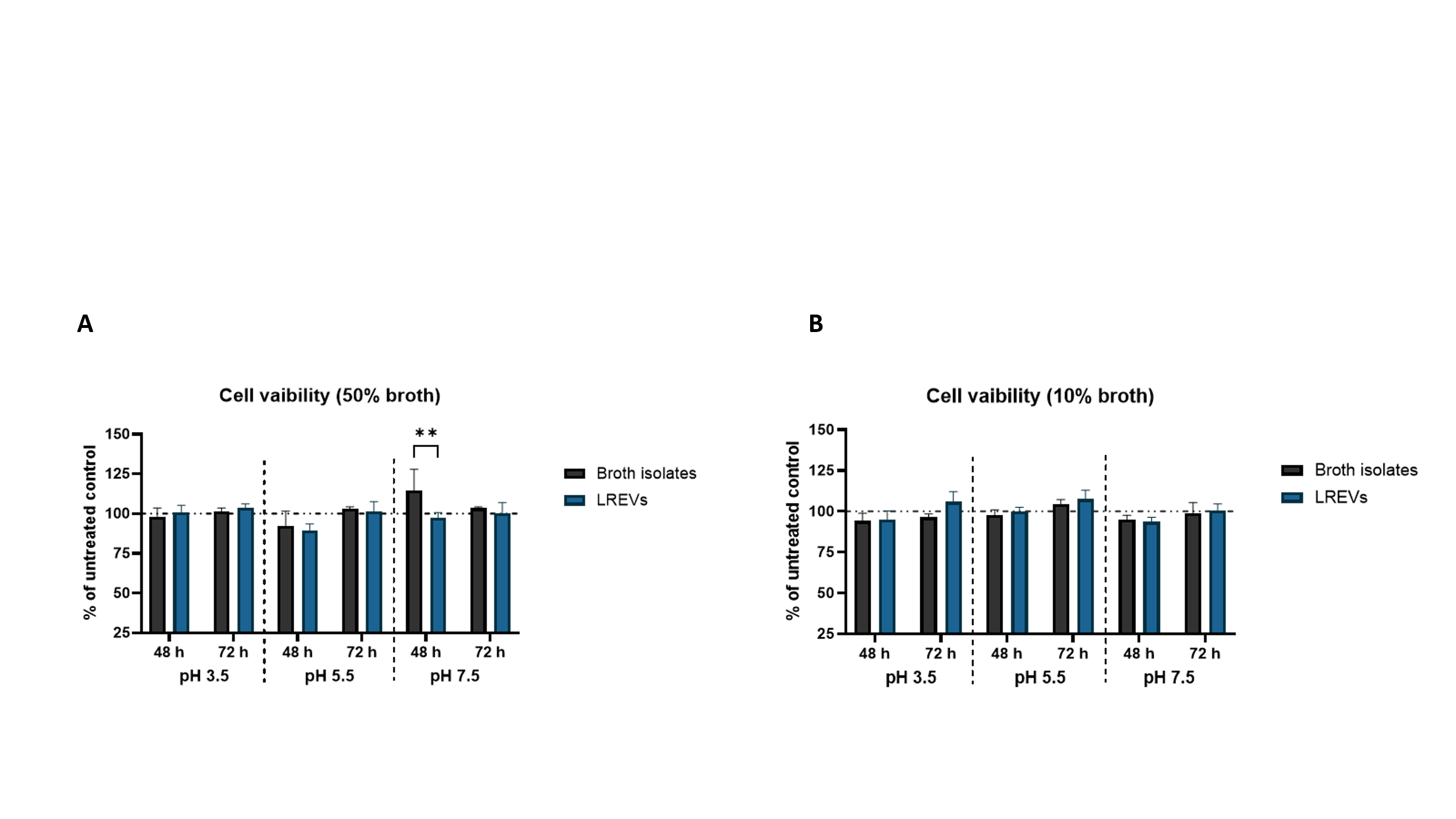
 **Supplementary Figure 5**. Cell viability of human epidermal keratinocyte (HEK) that treated by LREVs at concentration of 10^2^ EV per cells and broth isolates at the same volume/dilution ratio as LREVs including cell viability of HEK treated by LREVs and broth isolates that isolated from 50% of broth concentration (A) and 10% of broth concentration (B).

# Stability test

Method: We selected one type of EVs: LREVs, 50% broth concentration, pH 5.5, and 72-hour culture time. The stability of EVs was assessed by measuring size, size distribution and zeta potential for samples stored at 4°C for four weeks using single nanoparticle measurement system Exoid – Tuneable Resistive Pulse Sensing (Exoid, Izon Science, New Zealand). The advantage of using Exoid is that it provides a broader measurement range (50-330 nm using NP100 Nanopores) and includes zeta potential analysis using nanopore technology, offering detailed particle-by-particle analysis to assess aggregation and degradation.

Result: Our study confirmed that the EVs maintained the same size distribution profile, suggesting no aggregation or degradation during this period. In this context EVs were deemed stable. However, the zeta potential shifted from -13.7 mV before storage to -7.2 mV (Figure 1). This suggests that while the size and size distribution of our EV preparation are stable for at least four weeks at 4°C in PBS, the surface properties have been modulated by the current storage conditions. This indicates that additional storage buffers or alternative storage requirements are necessary.


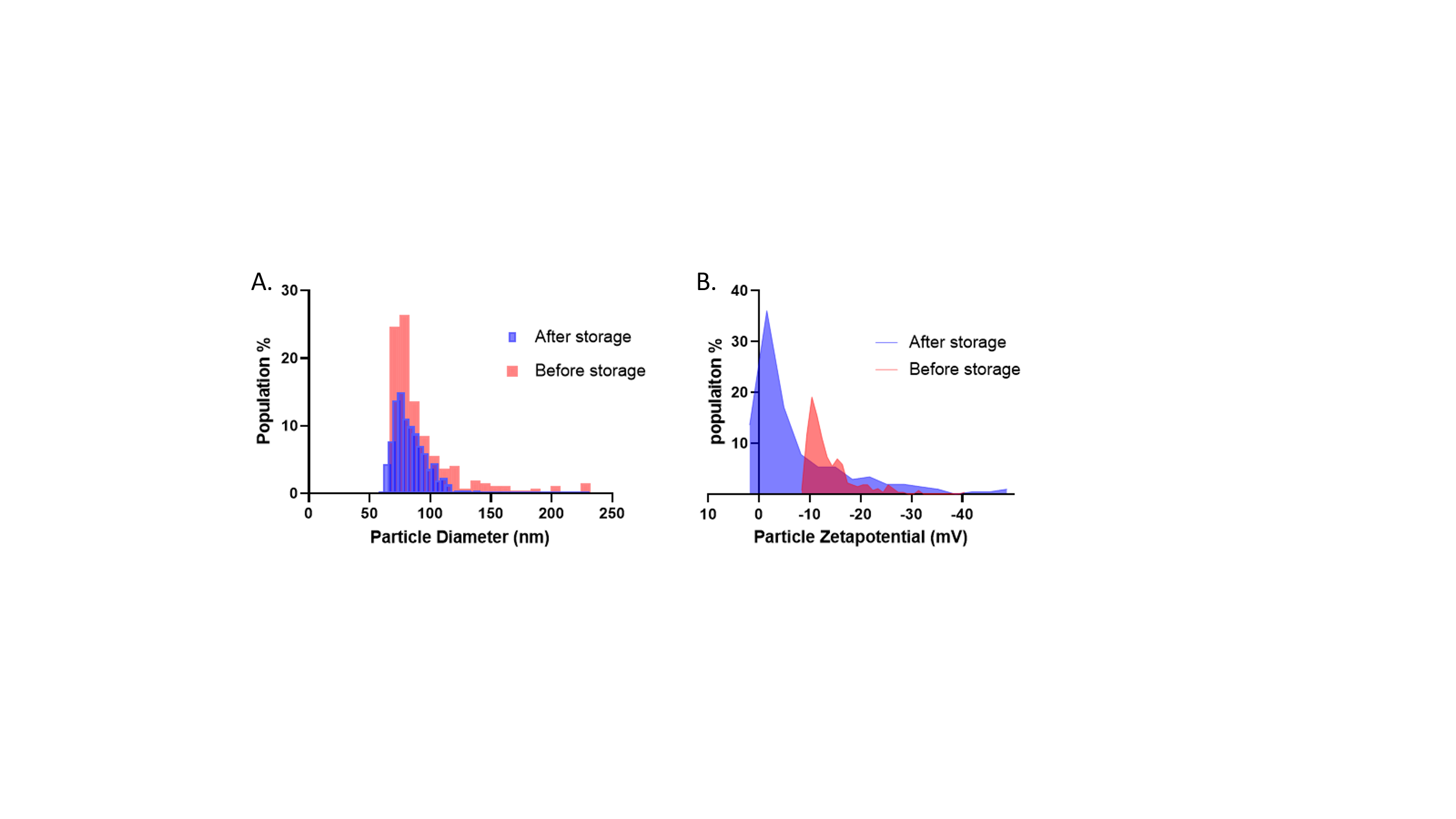


**Supplementary Figure 6**. (**A**) The size and size distribution of LREVs that isolated from 50% broth concentration, pH 5.5, and 72-hour culture time before/After storage for 4 weeks. (**B**) Zeta potential of LREVs that isolated from 50% broth concentration, pH 5.5, and 72-hour culture time before/After storage for 4 weeks.

**Supplementary** **Table 1**. The reduction of particle counts within LREVs obtained from modified culture conditions before and after Triton X-100 (TX) treatment.

| Broth concentration | pH | Samples | Particle counts | | Reduction (%) |
| --- | --- | --- | --- | --- | --- |
|  |  |  | Before TX | After TX |  |
| 50% | pH 3.5 | Broth isolates | 102 | 204 | -100 |
|  |  | LREV_48 h | 12061 | 1181 | 90 |
|  |  | LREV_72 h | 8288 | 310 | 96 |
|  | pH 5.5 | Broth isolates | 311 | 895 | -188 |
|  |  | LREV_48 h | 1876 | 667 | 64 |
|  |  | LREV_72 h | 6917 | 642 | 91 |
|  | pH 7.5 | Broth isolates | 339 | 640 | -89 |
|  |  | LREV_48 h | 527 | 1015 | -93 |
|  |  | LREV_72 h | 1611 | 893 | 45 |
| 10% | pH 3.5 | Broth isolates | 581 | 504 | 13 |
|  |  | LREV_48 h | 4145 | 887 | 79 |
|  |  | LREV_72 h | 1527 | 586 | 62 |
|  | pH 5.5 | Broth isolates | 52 | 343 | -560 |
|  |  | LREV_48 h | 5401 | 184 | 97 |
|  |  | LREV_72 h | 2526 | 192 | 92 |
|  | pH 7.5 | Broth isolates | 205 | 279 | -36 |
|  |  | LREV_48 h | 5444 | 208 | 96 |
|  |  | LREV_72 h | 6466 | 233 | 96 |
| Reduction (%) = (Before TX - After TX)/Before TX*100%  TX: Triton X-100 treatment | | | | | |
